# Screening for Drought Tolerance Within a Common Bean (*Phaseolus vulgaris* L.) Landrace Accessions Core Collection from the Lazio Region of Italy

**DOI:** 10.3390/plants13223132

**Published:** 2024-11-07

**Authors:** Enrica Alicandri, Ester Badiani, Anna Rita Paolacci, Emilio Lo Presti, Roberta Caridi, Roberto Rea, Francesco Pati, Maurizio Badiani, Mario Ciaffi, Agostino Sorgonà

**Affiliations:** 1 Department for the Innovation in Biological, Agro-Food and Forestry Systems, Via s. Camillo De Lellis, Tuscia University, I-01100 Viterbo, Italy; enrica.alicandri@unitus.it (E.A.); esterbadiani@gmail.com (E.B.); arpaolacci@unitus.it (A.R.P.); 2 Department of Agriculture, Mediterranean University of Reggio Calabria, Loc. Feo di Vito, I-89122 Reggio Calabria, Italy; emilio.lopresti@unirc.it (E.L.P.); roberta.caridi@unirc.it (R.C.); mbadiani@unirc.it (M.B.); asorgona@unirc.it (A.S.); 3 Regional Agency for the Development and the Innovation of Agriculture in Lazio, Via Rodolfo Lanciani 38, I-00162 Roma, Italy; r.rea@arsial.it (R.R.); f.pati@arsial.it (F.P.)

**Keywords:** *Phaseolus vulgaris* L. landraces, water stress, drought tolerance, yield, morpho-physiological traits

## Abstract

In the present work, a subset extracted from a core collection of common beans (*Phaseolus vulgaris* L.) landrace accessions from the Lazio region in central Italy was used to identify the most suitable drought-tolerant or -susceptible genotypes. By applying several morpho-physiological and agronomic selection criteria recommended by the available literature, we conducted a pre-screening experiment under controlled conditions on a primary group of 24 landraces. These landraces were chosen to represent the diversity in the Lazio region in terms of geographical provenance, elevation, landform, growth habit, customary water management in the field, and native gene pool. Pre-screening under controlled conditions allowed us to identify two subsets of landraces: one exhibiting the most drought-tolerance and one showing the most susceptibility to drought. These two subsets were then tested in field trials using two water treatments, i.e., normal irrigation versus no irrigation. Such field experiments were simultaneously conducted at two sites within the Lazio region, deliberately chosen to maximize their differences in terms of pedo-climatic conditions. This notwithstanding, the core findings from the two separate field experiments were remarkably consistent and coherent among each other, highlighting a notable degree of variability within the group of the tested landraces. In general, the morpho-physiological traits considered were found to be less responsive to water shortage than yield parameters. A statistically significant Landrace × Treatment interaction was found for almost all the yield parameters considered, suggesting that certain genotypes are more susceptible than others to water shortage. By taking into account the concept of “yield stability”, i.e., the ability to maintain comparatively high yields even under conditions of water scarcity, certain common bean landraces were found to be the most promising, in terms of drought tolerance. Two genotype pairs, in particular, could be of interest for studying the morpho-physiological and molecular mechanisms underlying drought tolerance in common bean, as well as for identifying quantitative trait loci associated with water scarcity, which could be beneficially employed in breeding programs. The results reported here also suggest that pre-screening under laboratory conditions, followed by targeted field trials, can constitute a reliable, efficient, and resourceful combined approach, in which morpho-physiological traits measured on juvenile plants might play a role in predicting drought tolerance at the agronomic level.

## 1. Introduction

Common bean (*Phaseolus vulgaris* L.) belongs to pulse crops indicated by the Food and Agriculture Organization of the United Nations as fundamental for human health, due to their high nutritional value and, above all, their role in the sustainable and environmental-friendly agriculture. In Europe, bean cultivation is predominantly carried out within the Mediterranean basin, with Italy cultivating more than 4000 Ha, yielding a production of over 10 thousand tons in 2023 [1].

Common bean is a crop species characterized by high genetic diversity, resulting in significant variability in plant architecture, seed traits, and yield potential [2,3], which allows its cultivation in a variety of environments and cropping systems [4]. In this regard, common bean is able to maintain its normal metabolic functions within reasonable limits of water scarcity [5]. Current climate scenarios consistently indicate a sharp variation in precipitation and temperature patterns, leading to an increasing frequency and intensity of drought stress episodes within the Mediterranean region. These changes negatively affect crop production as well as ecosystems’ structure and functions [6]. Furthermore, drought stress is the most impactful abiotic factors for bean cultivation and production [7]. Hence, in-depth studies on the interactions between common bean plants and drought stress at the morpho-physiological, molecular, germplasm and breeding levels are needed.

Drought resistance in plants encompasses a range of adaptive mechanisms, typically categorized into drought-escape, -avoidance, and -tolerance strategies [8,9]. In fact, common bean, a plant species with a high adaptive potential, employs various strategies for growth, development, and production in response to water scarcity. For instance, an enhanced mobilization of metabolic resources to the grain has been observed in bean genotypes [10,11,12], suggesting a drought-escape strategy. A drought-avoidance strategy was invoked for specific bean genotypes capable of increasing their root length density and extracting moisture from deeper soil layers [13,14]. Finally, a drought tolerance strategy, characterized by osmotic adjustments, increased cell elasticity, reduced cell size, and enhanced desiccation tolerance through protoplasmic resistance, has also been observed in selected bean genotypes [15,16]. Transcriptomics and genome-wide analyses have further confirmed the extensive variability of the mechanisms employed by bean plants in response to drought stress. For instance, numerous genes related to signal transduction, oxidative stress prevention, membrane transport, pathways associated to sulfur metabolism, root growth, and plant hormone signaling interactions, have been shown to be potentially involved in the tolerance of bean plants to water stress [17,18,19,20].

The extensive variability of molecular mechanisms and morpho-physiological responses to drought stress underscores the importance and utility of studying bean germplasm collections exhibiting a wide genetic variability, to evaluate and analyze their patterns of drought tolerance, in order to exploit specific morpho-physiological and molecular mechanisms evolved to adapt to peculiar agro-ecological contexts.

Crop landraces, broadly defined as dynamic populations of cultivated plants with historical origins, distinct identities, and lacking formal crop improvement, are typically characterized by a high level of genetic diversity. They are strongly adapted to local environmental conditions and associated with traditional farming systems, representing precious but often overlooked genetic resources for plant breeding programs, despite millennia of adaptation to stressful environments and specific pedo-climatic regions [21]. In Italy, the significant variability in terms of pedo-climatic contexts, coupled with historical and cultural fragmentation, has led to the development, over the centuries, of hundreds of bean landraces characterized by high morphological and genetic diversity, making them well suited to diverse agro-ecological settings [22,23,24,25,26,27,28]. However, to the best of our knowledge, the rich Italian heritage of bean landraces has not been thoroughly explored for the evaluation of their adaptive potential to climate changes, including drought tolerance, except in sporadic instances [29].

Conventional breeding for drought tolerance typically involves a screening phase to select elite genotypes for target traits. In the case of common beans, different screening approaches and methods have been utilized. These range from inducing drought stress using polyethylene glycol (PEG) on hydroponically grown plants under controlled conditions [29], to cultivating potted plants in greenhouse settings [30], to conducting field trials [31,32]. Taken alone, each of these approaches cannot escape the conundrum of reliability and reproducibility on one side, and adherence to the real world on the other side. Therefore, resorting to a combination of different screening strategies might be advisable. Furthermore, a pre-screening of drought-tolerant genotypes under controlled conditions, followed by testing the pre-selected genotypes in the field, would allow for a reduction in the number of plant tests. This would permit easier management and lower resources input for breeding programs.

Conventional breeding for drought tolerance in common bean has primarily relied on the selection of genotypes exhibiting superior performance in terms of grain yield under water stress, often in combination with yield aspects under well-watered conditions [12,33,34]. Little consideration, on the other hand, has been given to the morpho-physiological basis of both drought-dependent yield limitations and drought tolerance. Such understanding could guide and support common bean breeding programs, for instance, by combining parent lines exhibiting complementary morpho-physiological traits, with the support of Quantitative Trait Loci (QTL) analysis, to achieve an additive gene action towards drought tolerance [35].

Within the framework of an extensive and ongoing prospecting effort led by regional authorities to survey and exploit crops biodiversity and landraces heritage, the present study utilized a subset of common bean landraces derived from a core collection from the Lazio Region in Central Italy to identify the most suitable drought-tolerant or -susceptible genotypes. This was pursued by carrying out a pre-screening under controlled conditions, followed by field-testing of the pre-selected genotypes in two contrasting pedo-climatic contexts. Apart from practical and resource-saving implications, this combined approach also aimed to evaluate the impact of plants’ morpho-physiological traits on drought tolerance and their potential role as early predictors of agronomic performances under field conditions.

## 2. Materials and Methods

### 2.1. Pre-Screening for Drought Tolerance Under Controlled Conditions

#### 2.1.1. Plant Material

Twenty-four accessions of common beans (*Phaseolus vulgaris* L; Table S1 and Figure S1), each assigned to a distinct landrace [28], were selected from a core collection comprising 114 entries (accessions) representing 66 landraces from the Lazio Region. These accessions were collected from local farmers and conserved ex situ in the seed bank of the Regional Agency for Agricultural Development and Innovation of Lazio (ARSIAL, Rome, Italy), under the framework of Regional Law no. 15 dated 1 March 2000, titled “Protection of indigenous genetic resources of agricultural interest” (https://www.arsial.it/biodiversita/, accessed on 2 September 2024). The criteria adopted for extracting the 24 landrace accessions used in the present study from the entire germplasm collection were as follows (see Table S1):

1. provenance, covering a complete north-south transect within the Lazio Region.
2. Cropping pedo-climatic context, encompassing plateau, hill, and mountain landforms (from 380 to 969 m asl).
3. Growth habit, i.e., determinate versus indeterminate growth.
4. Customary water management during the growing season, i.e., irrigated *versus* rainfed.
5. Gene pool of origin, i.e., Mesoamerican or Andean.

The peculiarities of the 24 common bean accessions and their assignment to distinct landraces, evaluated through analyses of seed morphology, phaseolin and phytohemagglutinin patterns, and microsatellite loci, are summarized in Figure S2 and discussed in detail in a previous study of ours [28,36].

#### 2.1.2. Growth Conditions

The bean seeds, previously stored at 4 °C under vacuum, were surface sterilized for 2 min in 10% (*w*/*v*) NaOCl and germinated in the dark at 25 °C for 7 d in paper rolls soaked with 0.5 mM CaSO_4_. After germination, eight seedlings for each accession were selected for root length uniformity and then transferred into hydroponic units (four seedlings in each of 4.5-L glass pots) filled with an aerated nutrient solution [37], which was renewed every 2 days and maintained at pH 6.0 throughout the entire growth cycle. The growing units were kept in a growth chamber under a 14/10 h light/dark cycle (300 μE m^−2^ s^−1^ photosynthetic photon flux density at plants’ height), at 25/18 °C and 70% relative humidity.

#### 2.1.3. Imposing Drought Stress

Water stress was imposed to bean seedlings at their phenological stage V3 (first true leaf; approx. 12 days after germination; [38]) by gradually adding to the nutrient solution PEG 8000 (Sigma Aldrich catalog nr. P2139, St. Louis, MO, USA) until a final concentration of 6% (*w*/*v*). This was reached by three increments of 2% (*w*/*v*) each, supplied every two days, leading to a final osmotic potential of −0.6 MPa, according to the Michel’s equation [39]. Polyethylene glycol was omitted from the nutrient solution of control plants.

#### 2.1.4. Growth Parameters

At 0, 24, 48, 72, 96 and 168 h after reaching the final PEG concentration in the hydroponic solution, stem length (StL, cm), leaf area (LA, cm^2^), and leaf number (LN, n) were measured in each individual bean seedling. These measurements allowed the calculation of stem elongation rate (SER, cm d^−1^), leaf area expansion rate (LAER, cm^2^ d^−1^), and foliation rate (FoR, n d^−1^), respectively. Immediately after the final measurement (168 h), the bean seedlings were harvested. Their leaves and stems were separated among each other and weighted, both fresh and after drying in an oven at 70 °C, to obtain fresh and dry weights of leaves (LFW and LDW, g) and stems (SFW and SDW, g), respectively. The shoot dry weight (ShDW, g) was then calculated by summing LDW and SDW.

#### 2.1.5. Phenotypic Plasticity and Drought Stress Indexes

To screen drought-tolerant bean landraces, we took into account the different response coefficient (RC) as phenotypic plasticity index [40], the drought tolerance index (DTI; [41,42], and the drought tolerance efficiency (DTE; [43]). For each bean landrace, the RC was calculated as:

RC = V_C_/V_D_

where V_C_ and V_D_ are the average values of either SER, LAER, or FoR under control and drought stress conditions, respectively. RC values = 1 indicated no response to drought stress, whereas RC < 1 or RC > 1 indicated tolerance or susceptibility to drought stress, respectively.

For both the leaves and shoot of each landrace accession, the DTI and the DTE indexes were calculated as follows:

DTI = (DW_D_/DW_C_)/(ADW_D_/ADW_C_) DTE = DW_D_/DW_C_

where DW indicates the dry weight of each single landrace accession, whereas ADW indicates the average dry weight calculated from all the landrace accessions considered, the subscripts D or C stand for drought or control conditions, respectively.

#### 2.1.6. Statistical Analysis

The experimental design was completely randomized with four replicates for accession (A) and treatment (T). All data were tested for normality (Kolmogorov–Smirnoff test) and homogeneity of variance (Levene median test) and, when necessary, data were transformed.

All parameters were analyzed by two-way ANOVA with accessions and treatment level (control or drought stress) as main factors. Subsequently, Tukey’s test was used to compare the means of all parameters for each accession and treatment level.

For the RC values, the statistical significance was obtained by the probability level (*p* < 0.05) of the Accessions × Treatment (A × T) interaction [44].

Pearson correlation analysis was performed to test the correlations among the different drought indexes. SPSS Statistics software, v. 15.0 (IBM Corp., Armonk, NY, USA), was used for all the statistical analysis of the data.

### 2.2. Screening of Bean Landraces Under Field Conditions

#### 2.2.1. Plant Material

Nine common bean landrace accessions, characterized by opposite behavior towards water scarcity based on the pre-screening experiment above, were used to test their responses to drought during field experiments. In addition to the nine landrace accessions, two reference varieties, namely Borlotto Grecale and Cannellino di Atina, were used in the field experiments.

The Borlotto Grecale is a commercial variety with determinate growth and triple-purpose suitability (fresh market, dry beans, and industry), extensively cultivated in Italy, including the Lazio Region. It is favoured for its vigour, high pre-harvest defoliation tolerance, good fertility in terms of both pod number per plant and seed number per pod, and its adaptability to mechanical harvesting even at the waxy stage of maturity. The Cannellino di Atina is the only landrace from the Lazio Region that has received the Protected Designation of Origin (DOP) label, issued by the European Union.

#### 2.2.2. Experimental Sites and Design

Two field experiments were simultaneously conducted from June to October 2023 at two distinct experimental fields managed by ARSIAL. The sites where the two experimental fields are located are considered representative of contrasting pedo-climatic contexts for bean cultivation within the Lazio Region and Central Italy at large. The first site is located within the plain/coastal area of Cerveteri, belonging to the southernmost Maremma (41°59′26″ N; 12°05′26″ E; 31 m asl; average annual temperature 16.2 °C; average annual rainfall 928 mm). The second field site is located in the inland, high-hill area of Alvito, within the premises of the Abruzzo, Lazio and Molise National Park, at the slopes of the Apennines (41°40′12″ N; 13°44′87″ E; 482 m asl; average annual temperature 11.4 °C; average annual rainfall 1077 mm). These locations are referred to thereafter as Cerveteri and Alvito, respectively (Figure S1).

The main physic-chemical attributes of the soils at the two experimental sites are reported in Table S2.

A split plot randomized complete block experimental design (RCBD) with three replicates was implemented at both field sites. The main plots (34 × 16 m) were randomly assigned to the first experimental factor with two levels, namely adequate irrigation (Irrig) or no irrigation (NoIrrig), whereas the subplots (2.1 × 1.6 m) were randomly assigned to the second experimental factor, i.e., the common bean landrace accessions. Each sub-plot consisted of three rows, sown with a planting spacing of 0.3 m within the row and 1 m between the rows, for a total of 24 plants for each subplot. The main block was replicated three times.

#### 2.2.3. Cultivation

At both sites, sowing, as well as seedling and plant management, including thinning, were carried out according to the agronomic practices ordinarily adopted for common bean. This involved the use of plastic netting arranged over wooden pole structures, to support the climbing habit of the landrace accessions showing indeterminate growth.

The seeds of each landrace accession, including the commercial variety Borlotto Grecale, were sown at a depth of 5 cm on two sowing dates: June 1 for Alvito and June 11 for Cerveteri. At both sites, NPK 11-22-16 fertilizer was applied at pre-sowing at a rate of 200 Kg ha^−1^. There was no disease pressure during the trial, except for an incipient aphid infestation at Cerveteri, which was controlled by applying a deltamethrin-containing insecticide at the dose suggested by the manufacturer.

On-site weather stations at both Cerveteri and Alvito allowed to measure and record air temperature (°C), humidity (%), rain (mm), wind speed (m s^−1^), and solar radiation (W m^−2^) throughout the entire course of the field experiments.

#### 2.2.4. Imposing Drought Stress

Drought stress under field conditions was imposed on the plants by omitting irrigation throughout the entire plant cycle (denoted as NoIrrig plots hereafter), except for sporadic rescue drip irrigation applied when the volumetric soil water dropped below 15%, to ensure plant survival. Control plants, on the other hand, received adequate water via drip irrigation (denoted as Irrig plots hereafter), with the frequency and amount calibrated according to the plant phenological stage and to the evapotranspiration demand.

Volumetric soil water content (SWC, %; FieldScout TDR 350 Soil Moisture Meter, Spectrum Technologies, Inc., Aurora, IL, USA) was measured at both field sites during selected plant phenological stages (see below) at 12 sampling points across the drought-stressed field plots, and as many sampling points in the well-watered control plots.

#### 2.2.5. Morpho-Physiological Measurements

Morpho-physiological measurements on plants were carried out at three specific phenological stages, considered representative of vegetative or reproductive growth and known to be the most susceptible to drought stress [38]: stage V4, which begins when 50% of the plants have their third trifoliate leaf unfolded; stage R5, the pre-flowering stage, that begins when 50% of the plants show their first bud (determinate) or raceme (indeterminate); stage R8, the pod filling stage, which begins when 50% of the plants start filling their first pod, and the seeds inside the pod (10–12 cm in length) are discernible by sight and touch.

The three morpho-physiological parameters considered to be among the most responsive to drought and associated with drought tolerance in the studied plant species were the leaf relative water content (RWC; [45]), the leaf mass per area (LMA; [46]), and the net photosynthetic rate (NPR; [47]). The RWC (%) was determined for a healthy leaf in each subplot according to González and González-Vilar [48]. The LMA (g cm^−2^) was calculated as the ratio of leaf dry weight to leaf area [49]. The NPR (µmol CO_2_ m^−2^ s^−1^), along with stomatal conductance (SC, mol H_2_O cm^−2^ s^−1^) and transpiration rate (T, mmol H_2_O cm^−2^ s^−1^), were measured using a calibrated portable infrared gas analyzer (IRGA; mod. LI-6400; LI-COR, Inc.; Lincoln, NE, USA). This was set to a flow rate of 500 cm^3^ min^−1^, a leaf temperature of 26 °C, a CO_2_ concentration of 400 µmol (mol air)^−1^, and 1200 µmol m^−2^ s^−1^ of photosynthetically active radiation. Each measurement was carried out with a minimum and maximum wait time of 120 and 200 s, respectively, and matching the sample and the reference IRGAs to a 50 µmol (CO_2_) mol (air)^−1^ difference among each other before every change of plants. The leaf-to-air vapor pressure difference was set to 1.5 kPa and was maintained at a constant level by adjusting the humidity of the incoming air as needed. Measurements were carried out on two different plants from each subplot, using the central leaf of the third stage for the V4 stage, the last fully expanded leaf for the R5 stage, and the healthiest leaf near to the first pod for the R8 stage.

#### 2.2.6. Harvest

At commercial maturity [38], all the plants in each subplot were collected and the following yield parameters were measured: the total pods fresh weight per plot (FWP, g), the seeds yield per plot (SY, g), the 100-seeds weight (100SW, g), the number of pods per plant (NP, n.), and number of seeds per pod (NS, n.).

#### 2.2.7. Drought Stress Indexes

In coherence with the pre-screening experiment under controlled conditions (see above), the drought tolerance index was also calculated for the field experiments using all the morpho-physiological and yield parameters, as follows:

DTI (RWC) = (RWC_D_/RWC_C_)/(RWC_TD_/RWC_TC_) DTI (LMA) = (LMA_D_/LMA_C_)/(LMA_TD_/LMA_TC_) DTI (NPR) = (NPR_D_/NPR_C_)/(NPR_TD_/NPR_TC_) DTI (FWP) = (FWP_D_/FWP_C_)/(FWP_TD_/FWP_TC_) DTI (SY) = (SY_D_/SY_C_)/(SY_TD_/SY_TC_) DTI (100SW) = (100SW_D_/100SW_C_)/(100SW_TD_/100SW_TC_) DTI (NP) = (NP_D_/NP_C_)/(NP_TD_/NP_TC_) DTI (NS) = (NS_D_/NS_C_)/(NS_TD_/NS_TC_)

where the subscripts D or C indicate the values of the parameter of each landrace accession under drought or control conditions, respectively, whereas TD or TC indicate the corresponding average values calculated from all the landrace accessions considered.

Similarly, the drought tolerance efficiency (DTE) was calculated for each morpho-physiological and yield parameter as follows:

DTE (RWC) = RWC_D_/RWC_C_ DTE (LMA) = LMA_D_/LMA_C_ DTE (NPR) = NPR_D_/NPR_C_ DTE (FWP) = FWP_D_/FWP_C_ DTE (SY) = SY_D_/SY_C_ DTE (100SW) = 100SW_D_/100SW_C_ DTE (NP) = NP_D_/NP_C_ DTE (NS) = NS_D_/NS_C_

#### 2.2.8. Statistical Analysis

All the data were tested for normality (Kolmogorov–Smirnoff test) and homogeneity of variance (Levene median test) and, when necessary, data were transformed. All parameters were analyzed by two-way ANOVA with accessions and treatment level (control or drought stress) as main factors. The block (Bl) was also considered as a factor if the test detected a *p*-value < 0.05. Subsequently, Fisher’s test was used to compare the means of all parameters for each accession and treatment level (significance at *p* < 0.05).

The variability among the landrace accessions was estimated using the coefficient of variation (CV), which was categorized as low when less than 10%, moderate, i.e., 10–20%, or high, i.e., greater than 20% [50].

In order to verify the yield stability and drought tolerance screening across the two experimental sites, we conducted correlation tests (Spearman test) between the yield parameters and drought indexes obtained in Cerveteri and Alvito.

Further, a correlation test (Spearman test) was carried out to evaluate the relationship between DTI values calculated for shoot and leaf dry weight during pre-screening under controlled conditions and those calculated on the yield obtained from the field experiments.

Finally, in order to identify the morpho-physiological and yield parameters that serve as key predictors of a tolerance strategy against water stress, we applied a multivariate statistical approach (Principal Component Analysis, PCA) to datasets obtained from selected bean accessions, which were of particular interest in terms of tolerance or susceptibility to water stress during field experiments. PCA produced uncorrelated multivariate axes that could be interpreted as representing the adaptation of a given bean accession to water stress. Using the correlation matrix standardizes differences among variables caused by measurement scale. The importance of different traits on a given axis is indicated by the relative loading of the trait in the eigenvector.

The SPSS Statistics software, v. 15.0, was used for the statistical analysis of the data.

## 3. Results

### 3.1. Pre-Screening for Drought Tolerance Under Controlled Conditions

Three different approaches were used to identify tolerant common bean landrace accessions under simulated water stress in controlled conditions. The first approach considered the response coefficient (RC) index based on SER, LAER, and FoR, to measure the “phenotypic plasticity”. Indeed, depending on the sign and degree of the RC, information can be obtained about the “adaptive fitness” or “maladaptive fitness” of a given common bean landrace accession to water shortage. Table 1 shows that certain common bean landrace accessions exhibited a RC index clearly and significantly below 1, suggesting their drought tolerance, whereas other accessions had an RC clearly and significantly above 1, indicating their susceptibility to drought stress. The SER-based RC values, in particular, indicated the VE-0193, VE-0192, VE-0571, VE-0179, and VE-0378 as the most drought-tolerant landrace accessions, and VE-0473, VE-0110, VE-0277, VE-0215, and VE-0191 as the most susceptible ones. The LAER-based RC values indicated the VE-0222, VE-0378, VE-0472, VE-0273, and VE-0128 as the most drought-tolerant, and VE-0243, VE-0193, VE-0191, VE-0179, and VE-0183 as the most susceptible. Finally, the FoR-based RC values indicated VE-0459, VE 0378, VE-0277, VE-0110 and VE-0125 as the most drought-tolerant, and VE-0571, VE-0473, VE 0191, VE-0183, and VE-0179 as the most susceptible.

The second approach adopted for the screening of drought-tolerant common bean landrace accessions was based on using their shoot and leaf dry weights to calculate their drought tolerance indexes (DTI) and drought tolerance efficiencies (DTE), as defined above. According to Table 2, the DTI/DTE approach revealed a wide range of variability in stress susceptibility within the adopted germplasm collection. Based on such analysis, the VE-0277, VE-0459, VE-0224, VE-0117, and VE-0378 landrace accessions were considered drought-tolerant, whereas the VE-0261, VE-0191, VE-0193, VE-0183, and VE-0179 landrace accessions were regarded as drought-susceptible.

Pearson correlation analysis (Table S3) showed statistically significant correlations between the results of the RC approach (Table 1) and those of the DTI/DTE rankings (Table 2), in particular for the leaf-based RC values. Indeed, negative correlation between RC values and the DTI/DTE rankings indicated that the most drought-tolerant accessions showed the lowest RC values, thus suggesting that the two approaches can be reliably combined for the screening of drought-tolerant genotypes.

Hence, by considering together the results from phenotypic plasticity and from drought indexes (Table S4), the landrace accessions VE-0378, VE-0273, VE-0459, VE-0277, VE-0224, and VE-0117 could be classified as drought-tolerant, while the landrace accessions VE-0193, VE-0191, VE-0179, VE-0183, and VE-0261 could be considered as drought-susceptible.

The third approach adopted here to screen the present group of common bean landrace accessions for drought tolerance was based on the work of Ober et al. [42]. To understand the morpho-physiological basis of drought tolerance within germplasm collections belonging to the same plant species, they suggest focusing on genotype pairs that show similar yield potential under optimal water supply but contrasting DTI when exposed to drought stress. On one side, such approach allows to minimize the confounding effects of inherent intragroup variability of other factors, such as i.e., photosynthetic efficiency, photosynthates allocation, or nutrient utilization efficiency, which can influence the overall biological performance of the plants. On the other side, when applied to field conditions, this approach emphasizes the aspect of “yield stability”, that is, the ability of a given genotype to maintain satisfactory yield performance even in the presence of drought stress.

**Figure 1:**
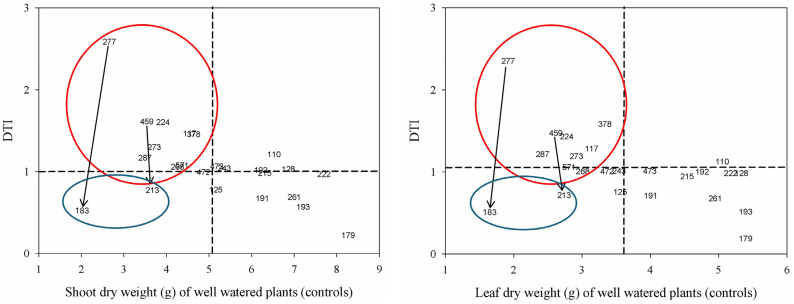
Screening common bean landrace accessions (indicated by abridged codes, see text) for drought tolerance on the basis of the “pairwise” approach proposed by Ober et al. [42]. DTI, drought tolerance index. The circles group bean landrace accessions showing similar dry biomass under adequate water availability (controls) but contrasting behavior under water stress (red, tolerant; blue, susceptible). The arrows indicate the bean accessions pairs showing similar biomass under control conditions but the widest difference in terms of drought tolerance index, upon exposure to water stress.

To apply the above approach, we plotted the shoot or the leaf dry weight of control (well-watered) plants of each common bean landrace accession against the respective DTI. This allowed grouping together, in a synoptic manner, genotypes (germplasm variants) showing similar features among each other’s (Figure 1). The results were consistent with those obtained using the RC and the DTI/DTE approaches (see Table 1 and Table 2, respectively), once again identifying the same group of common bean accessions as the most drought-tolerant in the growth chamber experiments.

These accessions were the VE-0277 (landform of origin: mountain; growth habit: indeterminate; customary water management: rainfed; see Table S1), the VE-0459 (hill; indeterminate; rainfed), the VE-0224 (hill; indeterminate; irrigated), the VE-0117 (plateau; determinate; irrigated), the VE-0378 (plateau; determinate; rainfed), the VE-0287 (mountain; indeterminate; irrigated), and the VE-0273 (mountain; determinate; irrigated). In contrast, the most drought-susceptible accessions were identified as the VE-0183 (hill; indeterminate; irrigated) and the VE-0213 (mountain; indeterminate; irrigated).

No obvious correlation pattern was found among tolerance/susceptibility to drought as assessed above, on one side, and certain of the landrace accessions’ characters reported in Table S1, namely the geographical provenance, the elevation, the growth habit, the customary water management, and the gene pool of origin, on the other side.

Based on the coherent results obtained from the above pre-screening approaches, we used the aforementioned nine bean landrace accessions for the subsequent field experiments. For the reasons given in Section 2.2.1, above, the commercial variety Borlotto Grecale, as well as the landrace Cannellino di Atina (VE-0110) were also included in the field experiments.

### 3.2. Screening Under Field Conditions

#### 3.2.1. Seasonal Growth at the Experimental Sites

The average values for maximum and minimum temperatures and humidity during the Cerveteri field experiment were 26.5/17.1 °C and 77.2%, respectively, with rainfall occurring over 26 days, totaling 100.1 mm rain (Figure S3).

Estimating volumetric soil water content at Cerveteri, coincident with the three plants’ phenological stages during which morpho-physiological measurements were carried out (V4, R5, and R8, see Section 2.2.5, above), indicated that SWC was never above 25% in all the plots (Figure S4). This suggests the occurrence of a generalized water shortage from the beginning of the plants’ vegetative growth onwards, probably due to the sandy nature of the soil at Cerveteri (Table S2). There were, however, evident differences in the intensity of water shortage between the Irrig and the NoIrrig plots (Figure S4), even though rain episodes, such as the one occurring the day before the SWC measurement at stage R5 (Figure S3), would have mitigated the differences between the two water regimes.

The average values for maximum and minimum temperatures, and humidity during the Alvito field experiment were 27.6/11.7 °C and 71.6%, respectively, with rainfall occurring over 57 days, totaling 314.0 mm of rain. Therefore, during the plants’ growing season, Alvito received three times more rainfall than Cerveteri (Figure S3). This, together with the water-retentive nature of the Alvito soil, unlike the Cerveteri soil (Table S2), helped in maintaining its volumetric water content always above 35%, irrespective of the Irrig/NoIrrig regimes applied to the experimental plots (Figure S4). Indeed, a statistically significant difference in SWC between the Irrig and NoIrrig plots in Alvito was only apparent at stage R8 of plants’ growth, although differences (not statistically significant) were also observed at the preceding stages.

Taken together, a higher soil water content at Alvito during the growing season and a wider temperature range, with lower night temperatures (Figure S3), would have caused a remarkably longer growing season at Alvito than at Cerveteri. This resulted in an increased duration of the vegetative period, asynchronous flowering, and a significant deferment in attaining the pods’ agronomic maturity (Table S5).

#### 3.2.2. Yield

Figure S5 shows the yield-related parameters measured at the end of the Cerveteri field experiment, whose ANOVA revealed a clear impact of water stress, resulting in a significant and generalised decrease (Table S6; *p* < 0.001). However, for three out of five yield parameters, namely FWP, SY, and NP, such negative impact varied depending on the landrace accession considered, as indicated by a significant accession × treatment interaction (*p* < 0.001). In particular, FWP and SY were significantly reduced by water scarcity in the VE 0287, VE-0273, VE-0213, and VE-0183 landrace accessions, as well as in the commercial variety Borlotto Grecale. A similar picture was also observed for NP, with the only difference that the reference landrace Cannellino di Atina was negatively affected by water stress, instead of Borlotto Grecale (Figure S5).

The results in Figure S5 were confirmed by calculating the drought tolerance indexes, namely DTI and DTE, based on the yield parameters (Table 3). Indeed, four out of five DTIs indicated the VE-0277, VE 0459, VE-0224, VE-0378, and VE-0117 as drought-tolerant accessions, whereas VE-0183, VE-0287, VE-0273, along with the two reference varieties Borlotto Grecale and Cannellino di Atina, were identified as drought-susceptible. Similar results were obtained when considering the other drought tolerance index, namely DTE (Table 3).

**Figure 2:**
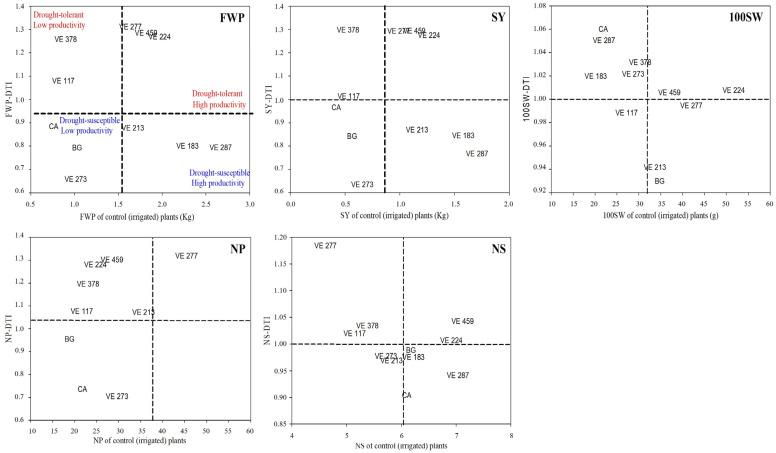
Screening common bean landrace accessions for drought tolerance at the Cerveteri field site on the basis of the “yield stability” approach proposed by Ober et al. [42]. DTI, drought tolerance index; BG, Borlotto grecale; CA, Cannellino di Atina. FWP, total fresh weight of pods; SY, seeds yield; 100SW, 100-seeds weight; NP, number of pods per plant; NS, number of seeds per pod. The dotted lines indicate the mean value for each yield parameter.

To validate the DTI/DTE-based selection of drought-tolerant landrace accessions, we applied the approach proposed by Ober et al. [42] to assess, as discussed above, the “yield stability” in the presence of drought stress. For such purpose, the yield-based DTIs of each landrace accession were plotted against the corresponding yield parameters obtained from irrigated (control) plots (Figure 2). It is evident that, for most of the yield parameters, the VE-0459, VE-0277 and VE-0224 landrace accessions can be classified as drought-tolerant and productive. Conversely, the two reference varieties, namely Borlotto Grecale and Cannellino di Atina, and the VE-0273 landrace accession can be considered as drought-susceptible and non-productive. Intermediate results were observed for the VE-0378 and VE-0117 landrace accessions, which resulted to be drought-tolerant but not productive, and for the VE-0213, VE-0183 and VE-0287 landrace accessions, which were found to be susceptible to water stress but highly productive under irrigated conditions (Figure 2).

The yield results obtained from the Alvito field experiment confirmed those obtained from the Cerveteri one. Indeed, water shortage affected all the yield parameters considered (Figure S6). Furthermore, a significant Accession × Treatment interaction was observed for 4 out of 5 of the yield parameters (Table S6). In particular, water stress reduced the FWP and the SY of the two reference varieties, as well as those of the VE-0183, VE-0213, VE-0273, and VE-0287 landrace accessions. Additionally, the VE-0117 had to be included into such group, when considering the NP parameter (Figure S6). The 100SW parameter was also reduced in common bean plants grown without irrigation, but only in the VE-0117 and the VE-0213 landrace accessions (Figure S6).

Most of the yield-related DTIs and DTEs calculated for the common bean plants at the Alvito field site indicated that the VE-0277, VE-0224, VE-0378, and VE-0459 landrace accessions could be regarded as drought-tolerant (Table 4). Conversely, the VE-0213 and VE-0117 landrace accessions (2 out of 5 parameters), as well as the VE-0183, VE-0273, and VE-0287 ones (4 out of 5 parameters), can be considered as drought-susceptible (Table 4).

**Figure 3:**
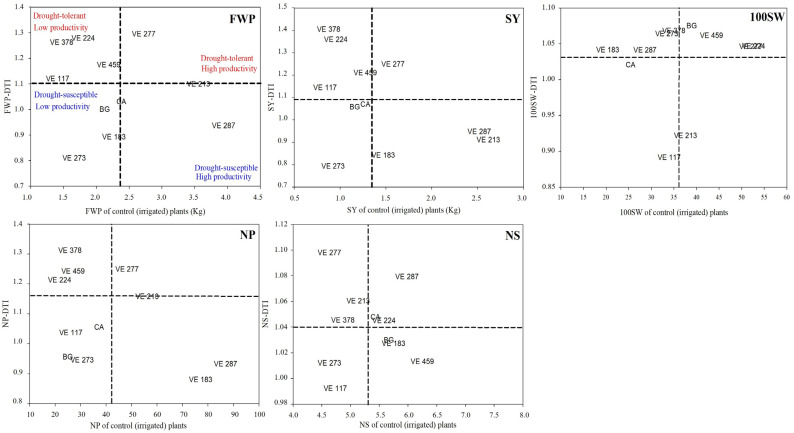
Screening common bean landrace accessions for drought tolerance at the Alvito field site on the basis of the “yield stability” approach proposed by Ober et al. [42]. DTI, drought tolerance index; BG, Borlotto grecale; CA, Cannellino di Atina. Acronyms for yield parameters as in Figure 2. The dotted lines indicate the mean value for each yield parameter.

When applying the “yield stability” approach proposed by Ober et al. [42] to the Alvito dataset (Figure 3), we found that, for most of the yield parameters considered, the VE-0378, VE-0459, and VE-0224 landrace accessions clustered into the drought tolerance/low productivity quadrant, whereas the VE-0213, VE-0183, and VE-0287 landrace accessions grouped into the drought susceptible/high productivity quadrant. The VE-0277 landrace accession was identified as both drought-tolerant and highly productive, whereas the remaining landrace accessions clustered into the drought-susceptible/low productivity quadrant.

**Figure 4:**
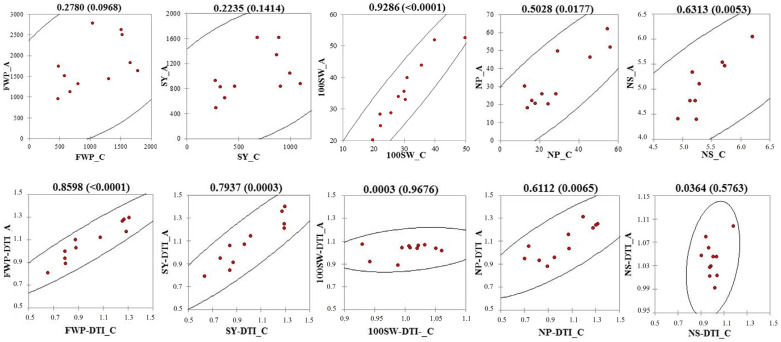
Correlations among the yield results obtained from bean landrace accessions (red dots) grown in the non-irrigated plots of Cerveteri (_C) or Alvito (_A). Upper row, yield parameters; lower row, yield-related drought tolerance indexes (DTIs). The numbers above each plot indicate the coefficient of determination and, within brackets, the probability values, calculated by the Spearmen test. Acronyms for yield parameters as in Figure 2.

To test the reliability and the robustness of the approaches used for screening drought-tolerant common bean landrace accessions, we examined the correlations among the yield parameters measured in the NoIrrig plots at the two field sites. Specifically, we correlated among each other both the Cerveteri’s and Alvito’s yield parameters (Figure 4, upper row) and the corresponding yield-related DTIs (Figure 4, lower row). Three out of five yield parameters, specifically 100SW, NP and NS, showed significant correlation between the Cerveteri and the Alvito fields. Although not statistically significant, the FWP and the SY parameters also exhibited positive correlation between the two sites. Furthermore, the DTIs values showed statistically significant correlation between the two sites, with coefficient of determination of 0.86, 0.97, and 0.61 obtained for the FWP, SY, and NP parameters, respectively.

In addition to its effectiveness in relating agronomically important traits, such as drought tolerance and productivity, between two different field sites, we tested the ability of the correlation approach described above to predict drought tolerance at an early crop stage. To do this, the yield-related DTIs obtained from the fields trials were plotted against the biomass-related DTIs, namely the shoot dry weight (ShDW) DTI and the leaf dry weight (LDW) DTI, obtained from the pre-screening experiment conducted in growth chamber (see above). Indeed, the DTI’s based on FWP, SY, NP, and NS obtained from the Cerveteri field experiment were significantly correlated with the ShDW-DTI and the LDW-DTI from the same common bean accessions grown under controlled conditions, showing coefficients of determination ranging between 0.44 and 0.75 (Figures S7 and S8). Similar correlation results were also obtained for the Alvito field experiment, with coefficients of determination ranging between 0.51 and 0.75 (Figures S9 and S10).

#### 3.2.3. Morpho-Physiological Traits During Plants’ Growth

During the field trials, we measured selected morpho-physiological traits in order to evaluate their relative contribution to drought tolerance in the studied common bean landrace accessions from the Lazio Region. Measurements were carried out at three plant phenological stages assumed to be among the most responsive to water shortage (see Section 2.2.5, above), namely stage V4 (third trifoliate leaf unfolded), stage R5 (pre-flowering), and stage R8 (pod filling), to identify the most drought-susceptible among them.

The results of the ANOVA indicated that at Cerveteri the effect of water stress on the leaf relative water content (RWC) was evident only at stage R5 (Table S7), with significant differences observed among the tested landrace accessions. Specifically, the accessions VE-0287 and VE-0273, as well as the reference variety Cannellino di Atina, exhibited a significant reduction in RWC under water stress conditions, unlike the other accessions (Figure S11, upper panel). Remarkably, in the case of VE-0183, there was even a higher RWC observed under water stress conditions, compared to the control (Figure S11). At the Alvito field, the RWC of the common bean landrace accessions was statistically affected by the irrigation regime at stage R5, but, unlike Cerveteri, the most pronounced effects were observed at stage R8 (Table S7), resulting in higher levels of RWC measured in the Irrig plots (Figure S11, lower panel). However, no significant differences in responses to the treatments among the common bean landrace accessions were highlighted (Accession × Treatment interaction *p* > 0.05, Table S7).

At the Cerveteri field site, the leaf mass per area (LMA) of the landrace accessions was statistically increased by the NoIrrig treatment (*p* < 0.01; Table S8; Figure S12). However, the Accession × Treatment interaction was found to be not significant (Table S8). At the Alvito field site, an increase of LMA in the NoIrrig plots was observed at stage V4 only (Table S8; Figure S12). Again, no statistically significant Accession × Treatment interaction was observed (Table S8).

In the Cerveteri field experiment, the net photosynthetic rate (NPR) of the common bean landrace accessions was statistically affected by water shortage (Table S9; Figure S13). However, different NPR responses to water regimes among the bean landrace accessions were observed at stage V4 only (*p* < 0.05 for the Accession × Treatment interaction, Table S9). In particular, the accession VE-0459 showed an increase in NPR under drought stress, unlike the commercial variety Borlotto Grecale, in which NPR was found to decrease; however, such difference disappeared from stage R5 onwards (*p* > 0.05 for the Accession × Treatment interaction, Table S9). Finally, stomatal conductance (SC) and transpiration rate (TR) at Cerveteri were affected by water regimes only during stage V4, with higher values in the NoIrrig plots (Table S9 and Figure S13).

In the Alvito field experiment, irrigated plants from most of the studied common bean landrace accessions showed a higher NPR than non-irrigated ones only at stage R5, but no statistically significant Accession × Treatment interaction was found (Figure S14; Table S10). Consistently, SC and TR values were also higher for most accessions in the Irrig plots than in the NoIrrig plots at both stages V4 and R5 (Figure S14; Table S10). However, the common bean landrace accessions showed different SC and TR responses to the water regimes at stage R5 only (*p* < 0.05 for the Accession × Treatment interaction, Table S10). In particular, water shortage decreased both SC and TR in the VE-0213, VE-0224, VE-0277, and VE-0287 accessions, as well as in the reference variety Cannellino di Atina (Figure S14).

To estimate the contribution of the morpho-physiological traits to overall drought tolerance, we correlated these traits to yield parameters under conditions of water shortage. In the Cerveteri field experiment, SC and TR showed a significantly positive correlation with FWP, SY, and NP at stage V4, whereas RWC showed a significantly positive correlation with SY at stage V4, and NPR with NS at stage R8 (Table 5, upper half). Conversely, in the Alvito experiment, significant correlations were mostly observed after the transition from the vegetative to the reproductive phase (Table 5, lower half): NPR, SC and TR were positively correlated with FWP and NS at stage R5, whereas SC and TR were positively correlated with FWP only at stage R8. Additionally, LMA was positively correlated with FWP, SY, and NS at stages R5 and R8, but only with FWP at stage V4. Finally, RWC was positively correlated with NS at stage V4.

Table S11 shows that the genetic variability within the nine common bean landrace accessions and the two reference varieties used here for the field trials is ample. By considering both morpho-physiological and yield parameters together, it was found that 56 out of 80 traits showed a coefficient of variation greater than 20%. Furthermore, the coefficients of variation under the NoIrrig water regime were higher than those under irrigated conditions (21 traits out 40).

#### 3.2.4. Principal Component Analysis (PCA) on Drought-Tolerant and Drought-Susceptible Common Bean Landrace Accessions

**Figure 5:**
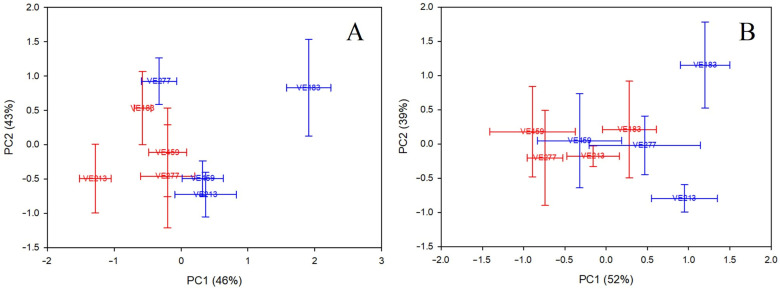
Scores (means and standard error bars) of the two first components (PC1 and PC2), which grouped the morpho-physiological and yield parameters of drought-tolerant (VE-0459 and VE-0277) and drought-susceptible (VE-0183 and VE-0213) common bean landrace accessions under the presence (red) or absence (blue) of water stress at the Cerveteri site (panel **A**) and Alvito site (panel **B**). The significant morpho-physiological and yield parameters are listed in Table S12. The proportion of explained variability is shown in parentheses.

Lastly, to understand the specific patterns of morpho-physiological and yield traits that differentiate drought-tolerant from drought–susceptible common bean accessions, we conducted a PCA on the entire dataset from both sites, using only two pairs of landrace accessions identified as drought-tolerant (VE-0459 and VE-0277) and drought-susceptible (VE-0183 and VE-0213). This made it feasible to reduce the morpho-physiological and yield traits from 20 to only five as relevant to explain the 89% and 91% of the total variability at the Cerveteri and Alvito sites, respectively (Table S12). The Kaiser-Meyer-Olkin Measure of Sampling Adequacy (0.636 for Cerveteri and 0.593 for Alvito) and the Bartlett’s Test of Sphericity (*p* < 0.001 for both sites) validated the PCA analysis. Furthermore, PCA grouped the five significant morpho-physiological and yield parameters into two components (PCs), which accounted for 46% (PC1) and 43% (PC2) of the variability for Cerveteri and for 52% (PC1) and 39% (PC2) of the variability for Alvito (Table S12). For the Cerveteri site, the parameters that most contributed to distinguish the groups were the yield parameters (FWP, SY, and NP), which fell into PC1, and the V4-SC and V4-TR, which fell into PC2 (Table S12). Figure 5 shows the biplot graph obtained by plotting each of the selected drought-tolerant and drought–susceptible common bean accessions under irrigated and non-irrigated conditions by means of their component scores. Notably, the drought-susceptible accessions, namely VE-0213 and VE-0183, were separated between the irrigated and non-irrigated conditions by PC1, while the drought-tolerant accessions, namely VE-0277 and VE-0459, were clustered together.

For the Alvito site, the gas exchange parameters at V4 and R5 were the most important morpho-physiological traits that determined the separation among the groups. Specifically, V4-SC and V4-TR fell into PC1, while the gas exchange parameters at R5 were included in PC2 (Table S12). Similar to Cerveteri, but even more distinctly, at the Alvito site the drought-susceptible accessions were separated between the irrigated and non-irrigated conditions by PC1, while the drought-tolerant accessions were grouped together (Figure 5).

## 4. Discussion

### 4.1. Identification of the Most Drought-Tolerant and -Susceptible Common Bean Landrace Accessions

Being local adaptations of domesticated species, landraces are known to be more productive than modern cultivars under conditions of low-input agriculture [51,52,53]. Furthermore, by incorporating ample genetic variability, they are also interesting genetic sources of traits for stress tolerance [54,55], yield stability [56,57], and seed nutrition [58,59]. As far as the common bean is concerned, several investigators have used its landraces as germplasm tools for screening drought tolerance [12,29,31,60,61].

In the present study, a selection of twenty-four landrace accessions from a much larger collection, previously gathered over several years in an ad hoc prospection of the Lazio territory carried out by ARSIAL, were first pre-screened in the growth chamber under controlled conditions. Then, nine of them, showing opposite responses in terms of drought tolerance, were tested in field trials. The common bean landrace accessions used in the field trials showed a high genetic variability among each other for several yield and morpho-physiological traits, suggesting that this germplasm pool could be of interest for plant breeding programs and a useful genetic tool for studying responses to drought stress in this important crop legume.

The pre-screening of common bean landrace accessions under controlled conditions was carried out by following three different approaches to select for drought tolerance conceptually robust. The first approach considered the “phenotypic plasticity” defined as “…the ability of a genotype to produce different phenotypic values for a given trait (character) in presence of different environmental conditions” [44] and calculated by the ‘response coefficient’ (RC) index [40] based on SER, LAER, and FoR parameters. Except for two landrace accessions, namely VE-0191 and VE-0378, for which all of the above parameters coherently indicated them as the most and the least susceptible to drought, respectively, the RC of the remaining common bean landrace accessions to water stress was much less univocal, especially regarding the stem and leaf parameters.

It is well known that different physiological mechanisms are put in place in the stem and in the leaf to resist water shortage. Indeed, the embolism resistance of the stem, due to an increased growth of the xylem tissues under drying conditions, allows a prolonged survival of plants during periods of water stress [62]. At the leaf level, water loss controlled by leaf shedding [63,64] and desiccation rate [65], stomata closure [66], and epidermal conductance [67] have been identified as traits related to drought avoidance. Hence, unlike most landrace accessions, the VE-0378, showing unequivocal responses to drought stress for all the parameters considered, could had used the drought tolerance strategies outlined above in a coordinated manner, which has been revealed to be very useful in controlling the highly complex plant water status [68]. Conversely, the VE-0191 landrace accession displayed the drought susceptible features.

The second approach used here for the pre-screening of drought-tolerant common bean landrace accessions under controlled conditions was based on the “drought indexes”, namely the drought tolerance index (DTI; [41,42]) and the drought tolerance efficiency (DTE; [43]). These indexes, by comparing yield in the presence or absence of water stress, focus on yield stability. We calculated the DTI and the DTE based on shoot and leaf dry weight, both considered reliable proxies of agronomic yield. Indeed, shoot biomass has been regarded as a useful and reliable trait for identifying drought tolerance in common bean genotypes [29,69,70]. Furthermore, differences among common bean genotypes under water stress conditions have been attributed to the accumulation of biomass in the shoot [69,70,71]. Finally, a strong positive correlation between shoot biomass and seed production has been observed among common bean genotypes under water scarcity conditions [70,71,72]. Based on the results obtained from the DTI/DTE approach, it was possible to identify a group of five drought-tolerant landrace accessions, and as many drought-susceptible ones. By integrating the results derived from the two approaches outlined above, namely phenotypic plasticity and drought indexes, two well-defined groups were identified: one of drought-tolerant landrace accessions, including VE-0378, VE-0273, VE-0459, VE-0277, VE-0224, and VE-0117, and another of drought–susceptible ones, including VE-0193, VE-0191, VE-0179, VE-0183, and VE-0261.

Unlike the previous “agronomy-based” approaches, the third approach used here for the pre-screening of common bean accession under controlled conditions was based on the physiological concept proposed by Ober et al. [42], who suggested that “...the genotype pairs that show similar yield potential but contrasted in DTI provide experimental material for dissection of morphological and physiological traits that confer drought tolerance…”. In other words, to target specific morpho-physiological and biochemical mechanisms of drought tolerance that determine the “efficiency” features, it is necessary to avoid those that provide higher yield/DTI/DTE independently of the water availability, as these contribute to the overall genetic potential and define the “superior” germplasm. This approach has also been used to identify P-efficient- [73] or N-efficient- [74] but also drought-tolerant- [29,75,76] genotypes in plant germplasm collections. According to the above criterion, the landrace accessions VE-0277, VE-0459, VE-0224, VE-0117, VE-0378, VE-0287, and VE-0273 belong to the drought-tolerant group, whereas the accessions VE-0183 and VE-0213 to the susceptible group. Such grouping aligns well with the results obtained from the application of the “agronomy-based” criteria mentioned above. Interestingly, the field trials conducted downstream of the pre-screening experiment in growth chamber revealed similar groupings for the common bean landrace accessions. Furthermore, on the basis of the “yield stability” concept proposed by Ober et al. [42] (see above), the accessions pairs VE-0277/VE-0459 and VE-0183/VE-0213 could be profitably employed for studying the morpho-physiological and molecular mechanisms underpinning drought tolerance in the present crop species.

No relationship was observed in the present work between the gene pool of origin of common bean accessions and their tolerance to drought. Miklas et al. [77] identified the germplasm of the Mesoamerican bean gene pool as a potential source of valuable drought-adaptive genes. The drought-tolerant landrace accessions from Lazio Region selected in the present investigation reveal different origins from one another [28]. Nevertheless, although the bean landrace accessions employed here could have derived from a single ancestral population, the indirect selection operated over time by farmers in a given area could have determined specific pedo-climatic adaptations.

### 4.2. Screening for Drought Tolerance During the Early Growth Stages

Screening genotypes for drought tolerance under controlled conditions at their early seedling stage can both save time and resources in the breeding programs [78], as well as avoid the interference of co-occurring biotic/abiotic challenges typically present under field conditions [79,80,81]. Hence, we tested here the assumption that responses to water stress observed in seedlings grown in the growth chamber could be reliable predictors of the behavior of mature plants grown in the open. To such aim, we used shoot dry weight- and leaf dry weight-based, or yield-based DTIs as proxies for drought-tolerance selection at seedling or mature stages, respectively. Indeed, strict and statistically significant correlations were found among the two different types of DTIs, suggesting the usefulness and convenience of pre-screening approaches under controlled conditions for breeding drought tolerance in the common bean crop. Furthermore, these results led us to speculate that the ability of a given genotype to accumulate photosynthates during the early vegetative stages could better sustain the future yield. Indeed, Ghanbari et al. [82] reported that the common bean genotypes COS16 and AND1007, which were found to be more efficient in accumulating nutrients in their aboveground tissues, also accumulated more reserves in their seeds under drought stress conditions. Similarly, a higher concentration of water-soluble carbohydrates has been found to determine drought tolerance in wheat [83,84].

### 4.3. Morpho-Physiological Traits Related to Drought Tolerance in Common Bean Landrace Accessions

Focusing on common bean genotype pairs that exhibit similar yield potential but opposite proneness to be impacted by water stress, *sensu* Ober et al. [42], could be a promising approach for unraveling the physiological basis of drought-related yield limitations. In addition to providing physiological selection tools for plant breeding programs [85], a detailed assessment of the morpho-physiological traits and mechanisms allows to combine parent genotypes with complementary traits, to achieve additive gene action for improving drought resistance [35,86]. In the present work, we investigated the genotypic variation of different drought-related morpho-physiological parameters, analyzed their responses to water stress across key phenological stages, and examined how these morpho-physiological responses correlate with yield traits, to understand their impact on agronomic performance under environmental stress.

The relative water content is an important indicator of leaf water status, as it reflects the balance between water supply to leaf tissues and transpiration rate [87]. In the present field trials, this parameter was less affected by the NoIrrig regime at Cerveteri compared to Alvito, with no differences among the common bean landrace accessions. In accordance with our results, the RWC of common bean plants can indeed be affected by water scarcity [60], but with poor and limited genetic variability [71].

The leaf mass per area is a trait widely used as a species-specific integrative indicator of the plant’s functional efficiency (photosynthetic and respiratory rates, composition chemistry, resistance to biotic and abiotic stresses [88,89]. In the present work, we observed an increase of LMA under water scarcity, more evident at Cerveteri than at Alvito. This increase could have been associated with a decrease in the leaf biomass allocated to the expansion of the leaf area. Such an adaptive strategy of course could be useful for decreasing the transpirating leaf surface under drought stress [90,91].

On the other hand, the mechanistic correlations among LMA and several compensatory responses deployed in plants against water deficit are all well established. These responses include anatomical traits, such as thick cell walls [92], major vein length per unit area [93], and the size/density of vein xylem conduits [94], as well as physiological traits, such as turgor loss point, extra-vascular and vascular hydraulic vulnerability, and the leaf water potential value inducing 50% loss (Ψ50; [91]).

The differences in the LMA responses to water scarcity between Cerveteri and Alvito, ranging from an increase at all stages in Cerveteri to barely affected throughout in Alvito, could have been due to significant differences in the water retention capacity of the respective soils. A soil water content permanently below 25% at Cerveteri, irrespective of the irrigation regimes applied, could have resulted in lower values of hydraulic-related physiological traits in the leaf, leading in turn to adaptive modification of anatomical and consequently morphological traits, such as an increase in LMA. It should be noted, however, that no statistically significant Accessions × Treatment interaction was found at either site, suggesting that probably LMA is a species-specific but not landrace (genotype)-specific functional trait.

The leaf photosynthetic system of common bean plants is severely damaged by water shortage [95,96,97]. Broadly speaking, our results confirmed the negative impact of water scarcity on the net photosynthetic rate (NPR) of common bean landrace accessions, which was more evident at Cerveteri than at Alvito. Indeed, a reduction in NPR was observed at both stage V4 (with a significant Accessions × Treatment interaction) and stage R8 in Cerveteri, but only at stage R5 in Alvito. It is known that the stress-related reduction of photosynthesis is a very complex effect, involving different physiological and molecular processes that can be grouped into stomatal- and non-stomatal-dependent mechanisms [98]. On average, stomatal conductance was found to decrease at stage V4 in Cerveteri, and at stages V4 and R5 in Alvito, correlating with the decrease in NPR and thus suggesting a stomatal-dependent limitation of photosynthesis. The plants at Cerveteri likely experienced more severe water scarcity than those at Alvito, as suggested by the SWC measurements (see above), although this was not reflected in changes in foliar RWC (see above). This supports the widely accepted notion that stomatal closure is more dependent on soil water availability rather than on leaf water status [99].

At Cerveteri, but not at Alvito, we noticed that, in general, plants in the NoIrrig plots had higher NPR than their irrigated counterparts at stage R5, which could have depended on the rain episodes that occurred a few days before the measurements. It has been reported that, upon re-watering of water-stressed plants, there is almost immediate re-opening of the stomata, and a recovery of NPR occurs [100], accompanied by an increase in the expression levels of photosynthesis- and hormone-related genes [101].

As far as individual landrace accessions are concerned, we found scant differences among them in terms of NPR and SC responses to water scarcity, appearing only at stage V4 in Cerveteri for NPR and only at stage R5 in Alvito for SC. In particular, we observed an increase in NPR in water-stressed VE-0459 plants and a decrease in SC in the VE-0213, VE-0224, VE-0277 and VE-0287 ones. Although the VE-0459 and the VE-0213 were found to be drought-tolerant and –susceptible landrace accessions, respectively, based on yield parameters, no clear difference was observed in terms of photosynthesis.

Finally, the phenological stages most affected by water stress in terms of photosynthetic function were V4 at Cerveteri and R5 at Alvito. These stage-specific effects of water stress could have reflected the relative preeminence of photosynthetic activity during the vegetative growth stage (V4) compared to the subsequent transition to flowering (R5 and R8), during which resource reallocation among the various plant organs predominates [102].

The importance of evaluating morpho-physiological traits and mechanisms underpinning the response to water scarcity clearly emerges when considering their implications and consequences for yield. Indeed, our results showed that in water-stressed common bean plants, the capacity to maintain comparatively high SC and TR values at stage V4 in Cerveteri and at stage R5 in Alvito was significantly correlated with comparatively high FWP, SY, NP, and NS. Consistent with our results, the ability to maintain a sufficient degree of stomata openness under water shortage, thereby allowing adequate biomass accumulation, is an adaptive strategy typical of the Tepary bean (*Phaseolus acutifolius* A. Gray), the most drought-tolerant among bean-related species [103].

Likewise, comparing the landrace accessions among each other revealed that adapting to drought stress by boosting an increase in LMA had a positive impact in terms of FWP, SY, and NS, albeit at Alvito only. By confirming previous results from others [90,91], this suggests that LMA is a functional trait whose accumulation is part of the drought-withstanding strategy adopted by common bean plants.

Considering that the univariate statistical analysis was unable to identify a unique morpho-physiological or yield pattern underlying the response of different bean accessions to water shortages, we conducted a PCA in search of efficient and meaningful “multi-trait classifiers” helpful in identifying the traits and mechanisms that, individually or in combination, enable plants to withstand water shortage, thereby conferring drought tolerance. Based on the morpho-physiological and yield responses of the individual common bean accessions to optimal or stressful conditions, the following considerations can be drawn:

(1) For the drought-tolerant common bean accessions, namely VE-0459 and VE-0277, PCA revealed that the gas exchange parameters at both the V4 and R5 stages were not altered by the NoIrrig treatment. Hence, mechanistically speaking, no substantial degree of stomatal closure occurred in response to water shortage, resulting in a sustained transpiration and preservation of the photosynthetic activity and yield, which was more evident at the Cerveteri site. However, in these bean accessions, it cannot be excluded that other morpho-physiological mechanisms may be employed to support the photosynthetic machinery and therefore maintain productivity under stressful conditions.
(2) For the drought-susceptible common bean accessions, namely VE-0213 and VE-0183, PCA revealed that at the Alvito site, irrigated plants were separated from the non-irrigated ones by gas exchange parameters at the R5 stage, which indicated behavioral differences in the presence and absence of water. In particular, stomatal opening was reduced, resulting in a decreased transpiration rate and photosynthetic activity. This reduction was one of the likely causes of decreased photosynthetic biomass and, therefore productivity. Indeed, common bean has been reported to be very sensitive to drought stress during flowering [97]
(3) At the Cerveteri site, PCA showed that for both the above drought-susceptible accessions, irrigated plants were separated from the non-irrigated ones by yield parameters, whereas no change in gas exchange was observed in response to water shortage. This suggests that other morpho-physiological mechanisms, apart from gas exchanges, may be negatively affected by water stress, resulting in poor growth and productivity. Among these mechanisms are shifts from primary to secondary metabolism and/or the redirection of assimilates toward the root system, at the expense of the aboveground vegetative and reproductive growth [104].

## 5. Conclusions

In the present work, a subset extracted from a core collection of common bean landraces from the Lazio Region, Central Italy, was used to identify the most drought-tolerant or -susceptible genotypes. By applying several morpho-physiological and agronomic selection criteria recommended by the available literature, we conducted a pre-screening experiment under controlled conditions on a primary group of 24 common bean landrace accessions. This allowed us to distinctly identify two subsets: one behaving as the most drought-tolerant, including the VE-0117, VE-0224, VE-0273, VE-0277, VE-0287, VE-0378, and VE-0459 landrace accessions, and one showing the most susceptibility to drought, comprising the VE-0183 and VE-0213 landrace accessions. These same two subsets were then tested in field trials using two water treatments, i.e., normal irrigation *versus* no irrigation. Such field experiments were simultaneously conducted at two sites within the Lazio Region, displaying contrasting pedo-climatic features among each other. Despite the deliberate selection of two field sites to maximize their differences in terms of pedo-climatic conditions, and although these differences did indeed have evident consequences in terms of overall agronomic performance and the duration of crop’s life cycle, the core findings from the two separate experiments were remarkably consistent and coherent among each other. The main results obtained are sketchily resumed below.

- The bean accessions used in field trials exhibited a remarkable degree of variability in response to the two different water regimes.
- The morpho-physiological traits considered were found to be less responsive to water shortage than yield parameters.
- A statistically significant Accession × Treatment interaction was found for almost all the yield parameters considered, suggesting that certain genotypes are more susceptible than others to water shortage. Based solely on drought tolerance indexes, the common bean accessions VE-0224, VE-0277, VE-0378, and VE-0459 were identified as drought-tolerant, whereas VE-0183, VE-0273, and VE-0287 were found to be susceptible to drought.
- When also considering the concept of “yield stability”, i.e., the ability to maintain comparatively high yields even under conditions of water scarcity, the bean accession VE-0277 was found to be the most promising, in terms of drought tolerance. However, VE-0224 and VE-0459 also exhibited satisfactory yield performances.
- The bean genotype pairs VE-0277/VE-0213 and VE-0459/VE-0183 could be considered of interest for studying the morpho-physiological and molecular mechanisms underlying drought tolerance in common bean. Additionally, they could be utilized to identify quantitative trait loci associated with water scarcity, which could be beneficially employed in breeding programs.
- Taken together, the results reported here also suggest that pre-screening under laboratory conditions, followed by targeted field trials, can constitute a reliable, efficient and resourceful combined approach, in which morpho-physiological traits measured on juvenile plants might play a role in predicting drought tolerance at the agronomic level.

## Figures and Tables

**Table 1:** Growth rates and phenotypic plasticity measured in common bean landrace accessions after simulated drought stress under controlled environmental conditions. C, control plants; D, drought-stressed plants; SER, stem elongation rate (cm d^−1^), LAER, leaf area expansion rate (cm^2^ d^−1^); FoR, foliation rate (n d^−1^); RC, response coefficient. The results of two-ways ANOVA are shown: A, accession; T, treatment. Statistical significance: * 0.05 < *p* < 0.01; *** *p* < 0.001; NS, not statistically significant. Mean values of stressed plants statistically different from their respective controls (*p* < 0.05; Tukey test) are marked in bold. Red or blue colors for RC values denote drought-tolerance or drought-susceptibility, respectively.

Accession	SER		LAER		FoR		SER-RC	LAER-RC	FoR-RC
	A 9.30 ***		A 4.49 ***		A 3.62 ***				
	*T 0.42 ^NS^*		T 28.57 ***		T 3.52 *				
	A × T 1.85 *		A × T 1.50 *		A × T 1.78 *				
	C	D	C	D	C	D			
*VE-0110*	1.77	0.78	15.34	11.04	0.81	1.13	2.27	1.39	0.72
*VE-0117*	1.23	1.63	21.26	17.57	0.85	0.74	1.81	1.21	1.14
*VE-0125*	3.12	2.66	20.36	12.40	0.45	0.58	1.99	1.64	0.77
*VE-0128*	1.18	0.70	9.38	7.89	0.78	0.62	1.67	1.19	1.27
*VE-0179*	2.63	5.71	6.89	**1.42**	2.70	**0.68**	0.46	4.85	5.05
*VE-0183*	0.28	0.27	3.90	6.82	0.96	0.35	1.04	8.46	2.76
*VE-0191*	6.01	**1.98**	13.23	**8.20**	2.04	**1.15**	4.26	3.53	1.76
*VE-0192*	0.49	**1.28**	30.51	**14.25**	1.02	0.89	0.38	2.14	1.15
*VE-0193*	1.36	**4.01**	27.31	**9.55**	0.89	0.59	0.34	3.21	1.50
*VE-0213*	5.08	5.98	13.61	7.22	0.52	0.76	0.85	1.89	0.81
*VE-0215*	9.22	**2.78**	17.39	**8.48**	1.50	1.88	3.32	2.05	0.80
*VE-0222*	3.47	5.70	11.78	13.26	1.89	1.94	0.60	0.64	0.97
*VE-0224*	5.77	4.18	13.83	8.73	1.44	1.04	1.38	1.58	1.39
*VE-0243*	4.63	3.85	17.55	**5.64**	1.58	1.49	1.20	3.11	1.06
*VE-0261*	11.77	10.55	20.50	**9.29**	1.00	0.97	1.12	2.21	1.03
*VE-0268*	7.52	4.64	9.17	6.25	1.37	1.06	1.62	3.05	1.29
*VE-0273*	1.81	1.88	14.73	13.60	1.03	0.95	0.96	1.08	1.09
*VE-0277*	9.12	**3.87**	8.75	6.13	1.37	2.18	2.36	1.43	0.63
*VE-0287*	4.73	4.00	7.76	4.65	1.43	1.01	1.18	1.67	1.42
*VE-0378*	0.87	1.56	7.81	11.53	0.44	0.80	0.56	0.68	0.55
*VE-0459*	6.04	9.19	16.06	12.44	0.79	1.53	0.66	1.29	0.51
*VE-0472*	2.02	2.26	20.92	23.53	0.97	0.76	0.89	0.89	1.27
*VE-0473*	1.38	1.39	26.95	**10.88**	1.42	0.86	2.21	2.48	1.64
*VE-0571*	1.71	3.74	10.11	7.17	2.01	1.25	0.45	1.90	1.55

**Table 2:** Rankings (descending order) of drought tolerance index (DTI) and drought tolerance efficiency (DTE) calculated from shoot dry weight (ShDW) or leaf dry weight (LDW) of common bean landrace accessions exposed to contrasting regimes of water availability under controlled environmental conditions. Red or blue colors for landraces accessions’ codes denote drought-tolerance or drought-susceptibility, respectively.

Landrace Accession	ShDW		Landrace Accession	LDW	
	DTI	DTE		DTI	DTE
**VE-0277**	2.59	213	**VE-0277**	2.35	194
**VE-0459**	1.61	133	**VE-0378**	1.58	130
**VE-0224**	1.60	132	**VE-0459**	1.47	121
**VE-0117**	1.46	120	**VE-0224**	1.43	118
**VE-0378**	1.45	120	**VE-0117**	1.28	106
VE-0273	1.30	107	VE-0287	1.22	100
VE-0110	1.21	100	VE-0273	1.19	98
VE-0287	1.17	96	VE-0110	1.12	92
VE-0571	1.07	88	VE-0571	1.06	87
VE-0473	1.06	87	VE-0243	1.01	83
VE-0268	1.06	87	VE-0473	1.01	83
VE-0243	1.04	86	VE-0472	1.01	83
VE-0128	1.03	85	VE-0268	1.01	83
VE-0192	1.02	84	VE-0192	1.00	82
VE-0472	0.99	82	VE-0128	0.98	81
VE-0215	0.98	81	VE-0222	0.98	81
VE-0222	0.97	80	VE-0215	0.94	78
VE-0125	0.78	64	VE-0125	0.75	62
VE-0213	0.77	63	VE-0213	0.72	59
**VE-0261**	0.69	56	**VE-0191**	0.71	58
**VE-0191**	0.67	55	**VE-0261**	0.67	56
**VE-0193**	0.57	47	**VE-0193**	0.51	42
**VE-0183**	0.50	41	**VE-0183**	0.48	40
**VE-0179**	0.22	18	**VE-0179**	0.19	15

**Table 3:** Rankings (descending order) of drought tolerance index (DTI) and drought tolerance efficiency (DTE) calculated from yield parameters, namely total fresh weight of pods (FWP), seeds yield (SY), 100-seeds weight (100SW), number of pods for plant (NP), and number of seed for pod (NS), in common bean landrace accessions exposed to contrasting regimes of water availability under field conditions at Cerveteri. Red or blue colors for the accessions codes of the landraces denote drought-tolerance or drought-susceptibility, respectively.

Accession	FWP		Accession	SY		Accession	100SW		Accession	NP		Accession	NS	
	DTI	DTE		DTI	DTE		DTI	DTE		DTI	DTE		DTI	DTE
VE-0277	1.31	93.22	VE-0378	1.30	88.02	Cannellino A	1.06	101.1	VE-0277	1.32	100.8	VE-0277	1.18	111.0
VE-0459	1.29	91.40	VE-0459	1.29	87.73	VE-0287	1.05	100.2	VE-0459	1.30	99.5	VE-0459	1.04	97.7
VE-0224	1.27	90.19	VE-0277	1.29	87.70	VE-0378	1.03	98.4	VE-0224	1.28	97.9	VE-0378	1.03	96.9
VE-0378	1.26	89.41	VE-0224	1.27	86.46	VE-0273	1.02	97.5	VE-0378	1.20	91.4	VE-0117	1.02	95.5
VE-0117	1.08	76.77	VE-0117	1.01	68.73	VE-0183	1.02	97.3	VE-0117	1.08	82.3	VE-0224	1.01	94.3
Cannellino A	0.88	62.84	Cannellino A	0.97	65.46	VE-0224	1.01	96.1	VE-0213	1.07	81.9	Borlotto G	0.99	92.6
VE-0213	0.88	62.40	VE-0213	0.87	58.96	VE-0459	1.01	95.9	Borlotto G	0.95	73.0	VE-0273	0.98	91.6
VE-0183	0.80	56.90	VE-0183	0.84	57.26	VE-0277	0.99	94.9	VE-0183	0.90	68.7	VE-0183	0.98	91.4
VE-0287	0.79	56.42	Borlotto G	0.84	57.12	VE-0117	0.99	94.3	VE-0287	0.83	63.2	VE-0213	0.97	90.8
Borlotto G	0.79	56.37	VE-0287	0.77	51.99	VE-0213	0.94	89.8	Cannellino A	0.74	56.3	VE-0287	0.94	88.2
VE-0273	0.66	46.77	VE-0273	0.63	43.05	Borlotto G	0.93	88.7	VE-0273	0.70	53.9	Cannellino A	0.90	84.7

**Table 4:** Rankings (descending order) of drought tolerance index (DTI) and drought tolerance efficiency (DTE) calculated from yield parameters of common bean landrace accessions exposed to contrasting regimes of water availability under field conditions at Alvito. Acronyms for yield parameters as in Table 3. Red or blue colors for accessions’ codes denote drought-tolerance or drought-susceptibility, respectively.

Accession	FWP		Accession	SY		Accession	100SW		Accession	NP		Accession	NS	
	DTI	DTE		DTI	DTE		DTI	DTE		DTI	DTE		DTI	DTE
VE-0277	1.30	92.10	VE-0378	1.40	95.24	Borlotto G	1.08	102.57	VE-0378	1.31	100.34	VE-0277	1.10	102.88
VE-0224	1.28	90.88	VE-0224	1.36	92.18	VE-0378	1.07	101.93	VE-0277	1.25	95.53	VE-0287	1.08	101.11
VE-0378	1.26	89.73	VE-0277	1.25	84.71	VE-0273	1.06	101.52	VE-0459	1.24	95.01	VE-0213	1.06	99.35
VE-0459	1.17	83.47	VE-0459	1.21	82.12	VE-0459	1.06	101.29	VE-0224	1.21	92.78	Cannellino A	1.05	98.16
VE-0117	1.12	79.66	VE-0117	1.14	77.61	VE-0224	1.05	99.87	VE-0213	1.16	88.54	VE-0378	1.05	97.95
VE-0213	1.10	78.17	Cannellino A	1.07	72.52	VE-0277	1.05	99.81	Cannellino A	1.06	80.69	VE-0224	1.05	97.91
Cannellino A	1.03	73.25	Borlotto G	1.06	71.78	VE-0183	1.04	99.38	VE-0117	1.04	79.26	Borlotto G	1.03	96.47
Borlotto G	1.00	71.15	VE-0287	0.95	64.28	VE-0287	1.04	99.36	Borlotto G	0.96	73.11	VE-0183	1.03	96.23
VE-0287	0.94	66.58	VE-0213	0.91	61.78	Cannellino A	1.02	97.38	VE-0273	0.95	72.29	VE-0459	1.01	94.93
VE-0183	0.89	63.45	VE-0183	0.84	57.10	VE-0213	0.92	87.98	VE-0287	0.93	71.28	VE-0273	1.01	94.82
VE-0273	0.81	57.60	VE-0273	0.79	53.79	VE-0117	0.89	85.07	VE-0183	0.88	67.28	VE-0117	0.99	92.96

**Table 5:** Pearson correlation analysis among morpho-physiological traits, measured at three growth stages, namely V4, R5, and R8 (first column from the left), and final yield parameters (first row from the top) of common bean landraces accessions grown at the two experimental field sites of Cerveteri and Alvito under water shortage. 100SW, 100-seeds weight; FWP, total fresh weight of pods; LMA, leaf mass area; NP, number of pods per plant; NPR, net photosynthetic rate; NS, number of seeds per pod; RWC, leaf relative water content; SC, stomatal conductance; SY, seeds yield; TR, transpiration rate. Statistically significant *p* values (<0.05) and coefficients of determination (*R*^2^) are in bold.

CERVETERI	FWP		SY		100SW		NP		NS	
	*p*	R^2^	*p*	R^2^	*p*	R^2^	*p*	R^2^	*p*	R^2^
V4-NPR	0.118	0.25	0.073	0.318	0.356	0.095	0.141	0.223	0.208	0.167
V4-SC	**0.020**	**0.490**	**0.013**	**0.542**	0.755	0.012	**0.035**	**0.417**	0.073	0.318
V4-TR	**0.009**	**0.583**	**0.006**	**0.625**	0.903	0.002	**0.016**	**0.516**	0.079	0.308
R5-NPR	0.979	8.2 × 10^−5^	0.704	0.016	0.989	8.2 × 10^−5^	0.653	0.024	0.903	0.002
R5-SC	0.410	0.074	0.426	0.069	0.624	0.027	0.871	0.003	0.978	8.2 × 10^−5^
R5-TR	0.548	0.040	0.586	0.033	0.724	0.014	0.989	8.2 × 10^−5^	0.539	0.044
R8-NPR	0.182	0.190	0.087	0.298	0.714	0.016	0.248	0.146	**0.031**	**0.440**
R8-SC	0.485	0.056	0.614	0.030	0.056	0.36	0.293	0.119	0.558	0.040
R8-TR	0.903	0.002	0.989	8.2 × 10^−5^	0.121	0.25	0.159	0.207	0.673	0.021
V4-LMA	0.860	0.004	0.818	0.007	0.673	0.021	0.765	0.010	0.129	0.241
V4-RWC	0.097	0.278	**0.035**	**0.417**	0.924	0.001	0.478	0.056	0.054	0.360
R5-LMA	0.968	0.000	0.860	0.004	0.129	0.241	0.724	0.014	0.273	0.132
R5-RWC	0.097	0.278	0.118	0.25	0.460	0.060	0.159	0.207	0.379	0.084
R8-LMA	0.468	0.060	0.214	0.167	0.410	0.074	0.100	0.278	0.087	0.297
R8-RWC	0.188	0.183	0.231	0.153	0.168	0.198	0.624	0.027	0.605	0.030
**ALVITO**										
V4-NPR	0.356	0.096	0.313	0.113	0.557	0.040	0.881	0.003	0.775	0.010
V4-SC	0.299	0.119	0.273	0.132	0.154	0.215	0.828	0.005	0.902	0.002
V4-TR	0.653	0.024	0.653	0.024	0.173	0.198	0.426	0.069	0.935	0.001
R5-NPR	**0.037**	**0.417**	0.056	0.360	0.129	0.241	0.807	0.007	**0.039**	**0.409**
R5-SC	**0.009**	**0.583**	0.005	0.640	0.0313	0.113	0.286	0.126	**0.034**	**0.426**
R5-TR	**0.009**	**0.583**	0.005	0.640	0.0313	0.113	0.286	0.126	**0.034**	**0.426**
R8-NPR	0.066	0.338	0.052	0.371	0.114	0.259	0.468	0.060	0.416	0.075
R8-SC	**0.040**	**0.405**	0.061	0.349	0.107	0.268	0.693	0.019	0.400	0.080
R8-TR	**0.040**	**0.405**	0.061	0.349	0.107	0.268	0.693	0.019	0.400	0.080
V4-LMA	**0.048**	**0.382**	0.163	0.207	0.776	0.010	1.00	0.000	0.252	0.144
V4-RWC	0.173	0.198	0.061	0.349	0.443	0.065	0.071	0.328	**0.015**	**0.527**
R5-LMA	**0.015**	**0.529**	**0.007**	**0.611**	0.807	0.007	0.061	0.349	**0.003**	**0.698**
R5-RWC	0.724	0.014	0.724	0.014	0.306	0.113	1.000	0.000	0.565	0.037
R8-LMA	**0.003**	**0.699**	**0.002**	**0.730**	0.892	0.002	0.100	0.278	0.034	0.426
R8-RWC	0.673	0.021	0.327	0.107	0.090	0.288	0.004	0.669	0.967	0.001

## Data Availability

The data contained within the present article and in its Supplementary Materials are freely available upon request to the corresponding author.

## Supplementary Materials

The following supporting information can be downloaded at: https://www.mdpi.com/article/10.3390/plants13223132/s1, Figure S1. Map of the municipalities (red dots) within the Lazio Region (Central Italy) where the accessions of the common bean (*Phaseolus vulgaris* L.) landraces were collected (prospection carried out by the Lazio Regional Agency for Development and Innovation in Agriculture, ARSIAL). Figure S2. Genetic relationships among the 24 common bean accessions from the Lazio Region screened for drought tolerance in the present experiments. Figure S3. Main meteorological parameters during the field experiments at the Cerveteri and Alvito sites. Figure S4. Volumetric soil water content (%) measured at the Cerveteri and the Alvito field sites, coincident with the three plants’ phenological stages described in the Material and Methods section. Figure S5. Yield parameters of common bean landrace accessions grown at the Cerveteri field site under contrasting irrigation regimes. Figure S6. Yield parameters of common bean landrace accessions grown at the Alvito field site under contrasting irrigation regimes. Figure S7. Correlation (Pearson test) between the drought tolerance index (DTI) based on the shoot dry weight (ShDW) of bean landrace accessions grown in growth chamber (pre-screening experiment; x axes) and the DTIs based on the yield parameters obtained from the same bean landrace accessions grown in the field at Cerveteri (y axes). Figure S8. Correlation (Pearson test) between the drought tolerance index (DTI) based on the leaf dry weight (LDW) of bean landrace accessions grown in growth chamber (pre-screening experiment; x axes) and the DTIs based on the yield parameters obtained from the same bean landrace accessions grown in the field at Cerveteri (y axes). Figure S9. Correlation (Pearson test) between the drought tolerance index (DTI) based on the shoot dry weight (ShDW) of bean landrace accessions grown in growth chamber (pre-screening experiment; x axes) and the DTIs based on the yield parameters obtained from the same bean landrace accessions grown in the field at Alvito (y axes). Figure S10. Correlation (Pearson test) between the drought tolerance index (DTI) based on the leaf dry weight (LDW) of bean landrace accessions grown in growth chamber (pre-screening experiment; x axes) and the DTIs based on the yield parameters obtained from the same bean landrace accessions grown in the field at Alvito (y axes). Figure S11. Leaf relative water content (RWC) in three phenological stages of bean landrace accessions grown under contrasting irrigation regimes at the two field sites of Cerveteri and Alvito. Figure S12. Leaf mass area (LMA) at three phenological stages of bean landrace accessions grown under contrasting irrigation regimes at the two field sites of Cerveteri and Alvito. Figure S13. Net photosynthetic rate (NPR), stomatal conductance (SC) and transpiration rate (TR) in three phenological stages of bean landrace accessions grown under contrasting irrigation regimes at the Cerveteri field site. Figure S14. Net photosynthetic rate (NPR), stomatal conductance (SC) and transpiration rate (TR) in three phenological stages of bean landrace accessions grown under contrasting irrigation regimes at the Alvito field site. Table S1. Common bean (*Phaseolus vulgaris* L.) landraces from the Lazio Region (Central Italy) screened for drought tolerance. Table S2. Soil analysis before seed sowing (0-30 cm depth) at the experimental field sites of Cerveteri and Alvito. Table S3. Pearson correlation analysis among different drought tolerance indexes calculated for common bean landrace accessions grown under controlled environmental conditions. Table S4. Comparison of rankings of different drought tolerance indexes calculated for common bean landrace accessions grown under controlled environmental conditions. Table S5. Comparative timetable of the key plant stages during the field experiments in Cerveteri and Alvito. Table S6. Two-ways ANOVA on the yield parameters from common bean landrace accessions grown at the Cerveteri and the Alvito field sites under contrasting irrigation regimes (treatments). Table S7. Two-ways ANOVA on the relative water content of common bean landrace accessions grown at the Cerveteri and the Alvito field sites under contrasting irrigation regimes (treatments). Table S8. Two-ways ANOVA on the leaf mass per area of common bean landrace accessions grown at the Cerveteri and the Alvito field sites under contrasting irrigation regimes (treatments). Table S9. Two-ways ANOVA on the leaf gas exchange parameters (NPR, net photosynthetic rate; SC, stomatal conductance; TR, transpiration rate) of common bean landrace accessions grown at the Cerveteri field site under contrasting irrigation regimes (treatments). Table S10. Two-ways ANOVA on the leaf gas exchange parameters (NPR, net photosynthetic rate; SC, stomatal conductance; TR, transpiration rate) of common bean landrace accessions grown at the Alvito field site under contrasting irrigation regimes (treatments). Table S11. Coefficients of variation of different traits [related to yield (Y), physiology (P), or morphology (M)] measured at three different growth stages, namely V4, R5, and R8, in common bean landrace accessions grown at two different field sites (C, Cerveteri; A, Alvito) under contrasting water regimes, namely irrigated (Irrig) or non-irrigated (NoIrrig).
