# Supplementary material for: Screening for Drought Tolerance Within a Common Bean (Phaseolus vulgaris L.) Landrace Accessions Core Collection from the Lazio Region of Italy
Source: Plants (Basel). 2024 Nov 7;13(22):3132. doi: 10.3390/plants13223132 (PMC11597768; doi:10.3390/plants13223132)
Supplement: Supplementary file 1 [file plants-13-03132-s001.zip › plants-3252705-supplementary.pdf]

Supplementary Figures

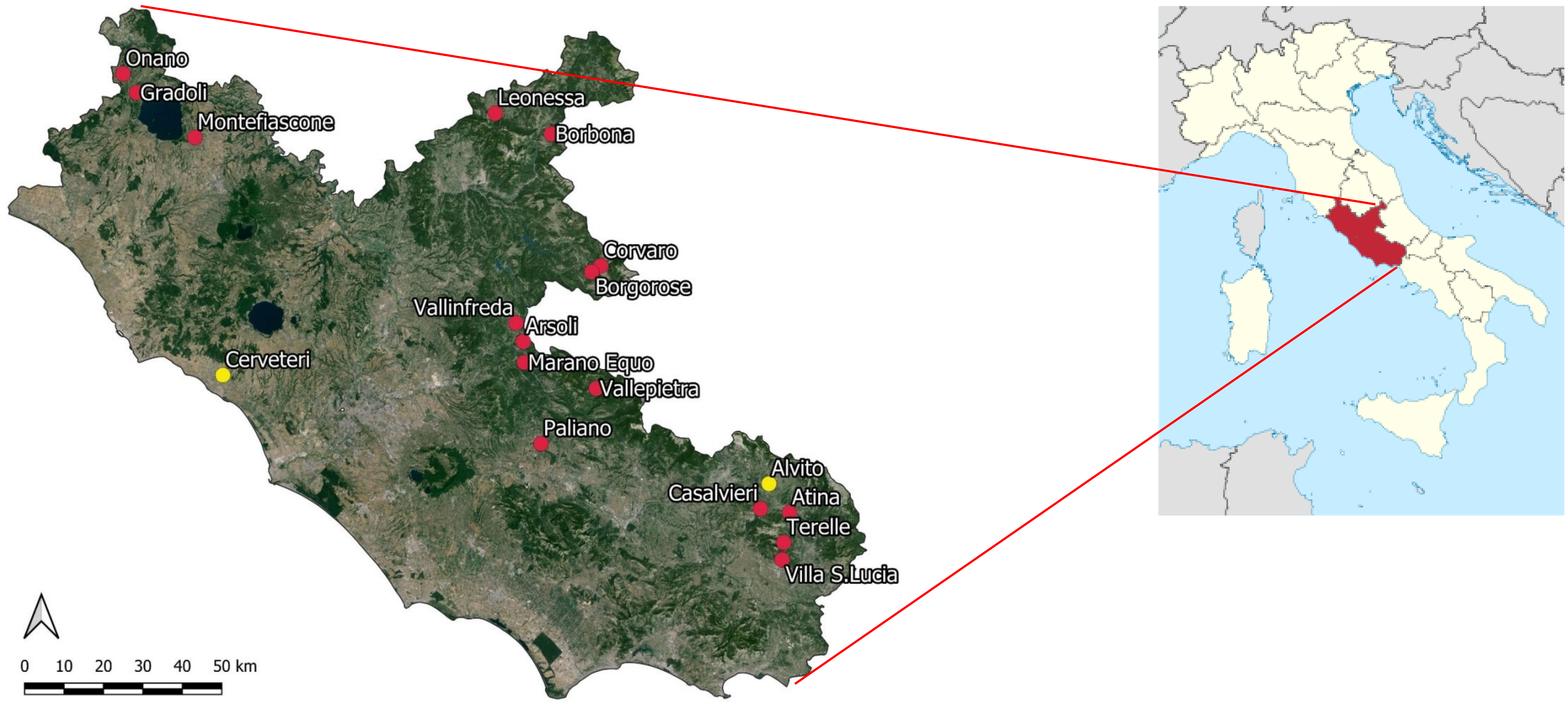

Figure S1. Map of the municipalities (red dots) within the Lazio Region (Central Italy) where the accessions of the common bean (Phaseolus vulgaris L.) landraces were collected (prospection carried out by the Lazio Regional Agency for Development and Innovation in Agriculture, ARSIAL). From north to south: **Onano**: Ciavattone piccolo (ARSIAL code VE-0179), Verdolino (VE-0191); **Gradoli**: Fagiolo del Purgatorio (VE-0183); **Leonessa**: Regina di Leonessa (VE-0277); **Montefiascone**: Verdolino di Montefiascone (VE-0192), Occhietto di Montefiascone (VE-0193); **Borbona**: Borbontino (VE-0261), Pelone (VE-0268); **Corvaro**: Fagiolo di Corvaro (VE-0273); **Borgorose**: Giallo di Nazzareno (VE-0472), Borlotto di Concetta (VE-0473);**Vallinfreda**: Cioncone (VE-0243); **Arsoli**: Fagiolina Arsolana (VE-0571); **Marano Equo**: Regina di Marano Equo (VE-0224); **Vallepietra**: Cappellette di Vallepietra (VE-0213), Pallino di Vallepietra (VE-0215), Romanesco di Vallepietra (VE-0222); **Paliano**: Fagiolo a Suricchio (VE-0459); **Casalvieri**: Cannellino con la mosca (VE-0378); **Atina**: Cannellino di Atina (VE-0110); **Terelle**: Bottoncino di Terelle (VE-0287); **Villa S. Lucia**: Cannellino Rosso di Piumarola (VE-0117), Cannellino Bianco di Piumarola (VE-0125); Cannellino Grigio di Piumarola (VE-0128). The two experimental field sites of **Alvito** and **Cerveteri** are also indicated (yellow dots). NOTE: map lines delineate study areas and do not necessarily depict accepted national boundaries.

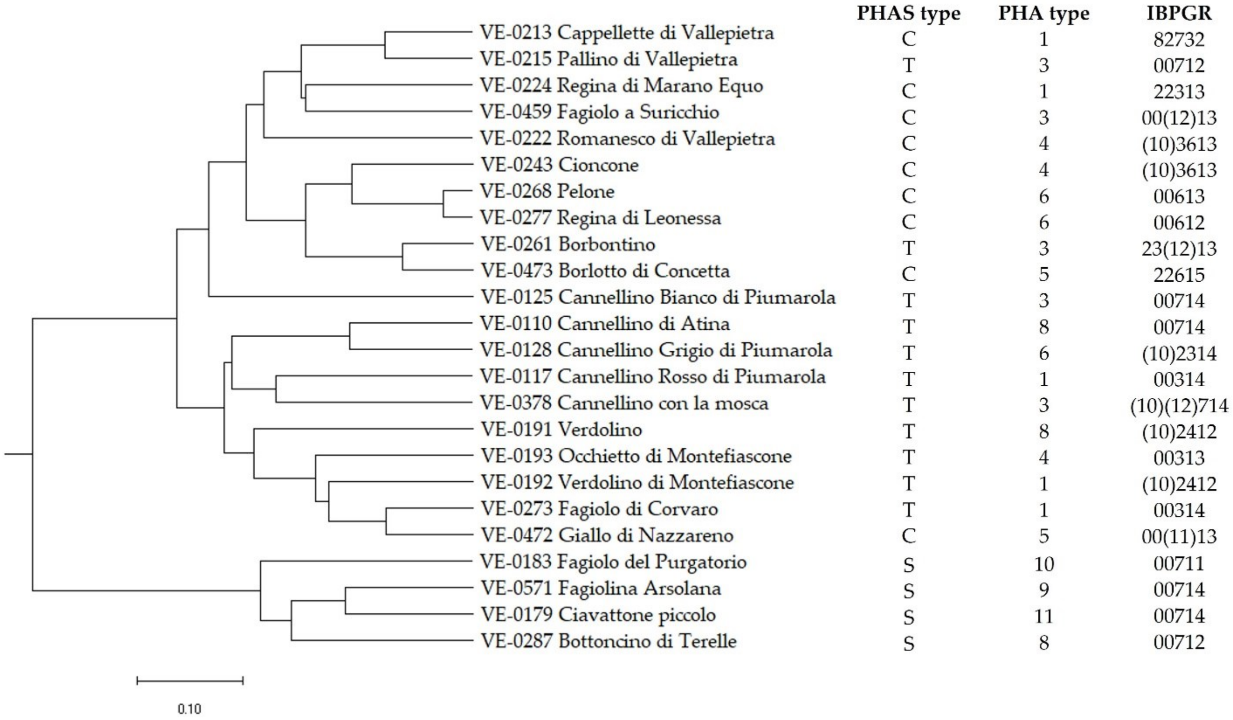

Figure S2. Genetic relationships among the 24 common bean accessions from the Lazio Region screened for drought tolerance in the present experiments. The dendrogram was built from the Nei's coefficient [36] and the unweighted pair group method with arithmetic mean (UPGMA) cluster analysis, by exploiting 12 simple sequence repeats (SSR) loci. For each bean accession, the phaseolin (PHAS), and phytohemagglutinin (PHA) types found in the seeds, as well as the International Board for Plant Genetic Resources (IBPGR) code indicating the seeds morphotype, are also reported. Further details in Catarcione et al. [28].

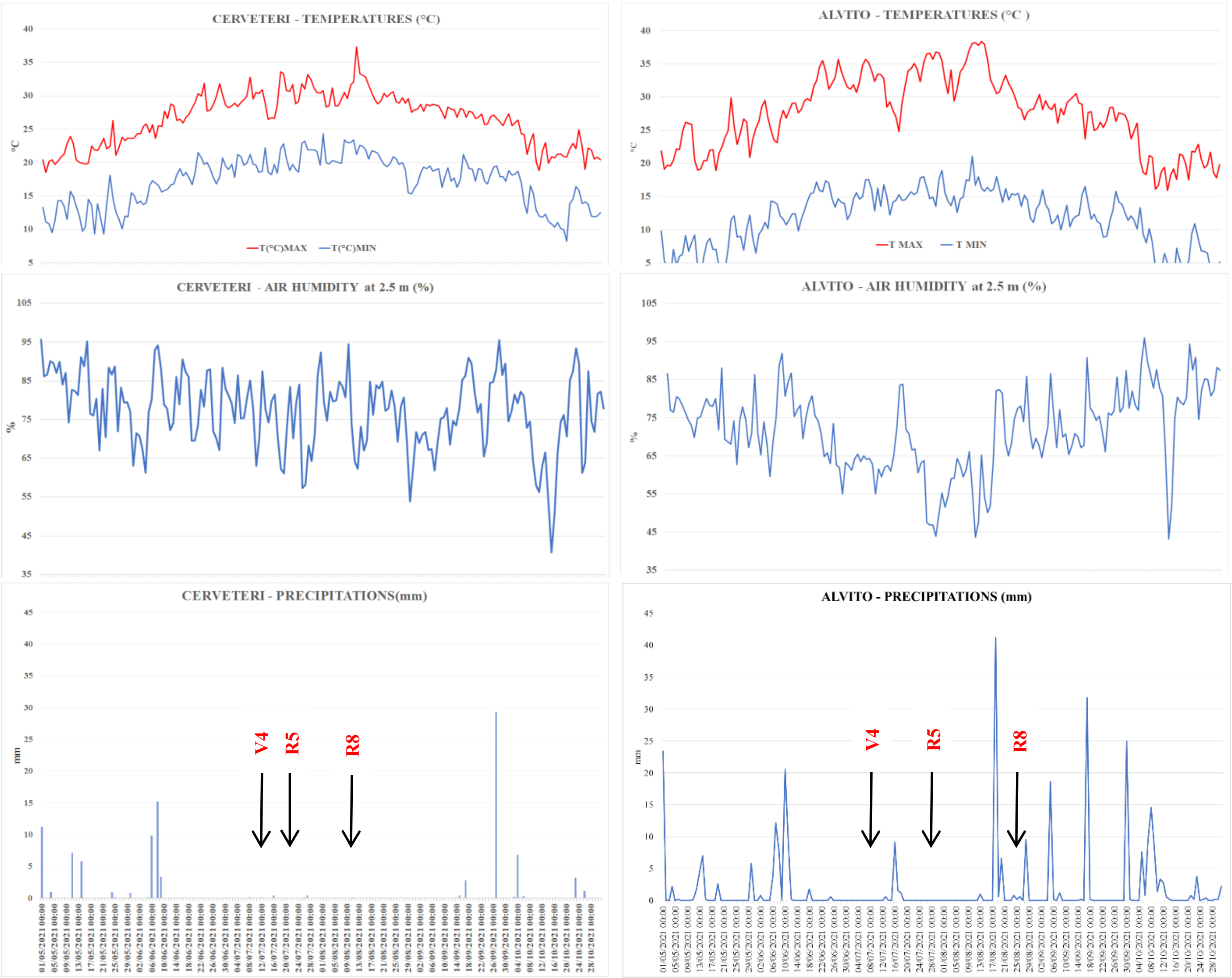

Figure S3. Main meteorological parameters during the field experiments at the Cerveteri and Alvito sites. The occurrences of the three plants' phenological stages during which morpho-physiological measurements were carried out (V4, R5, and R8) are indicated in the bottom panels.

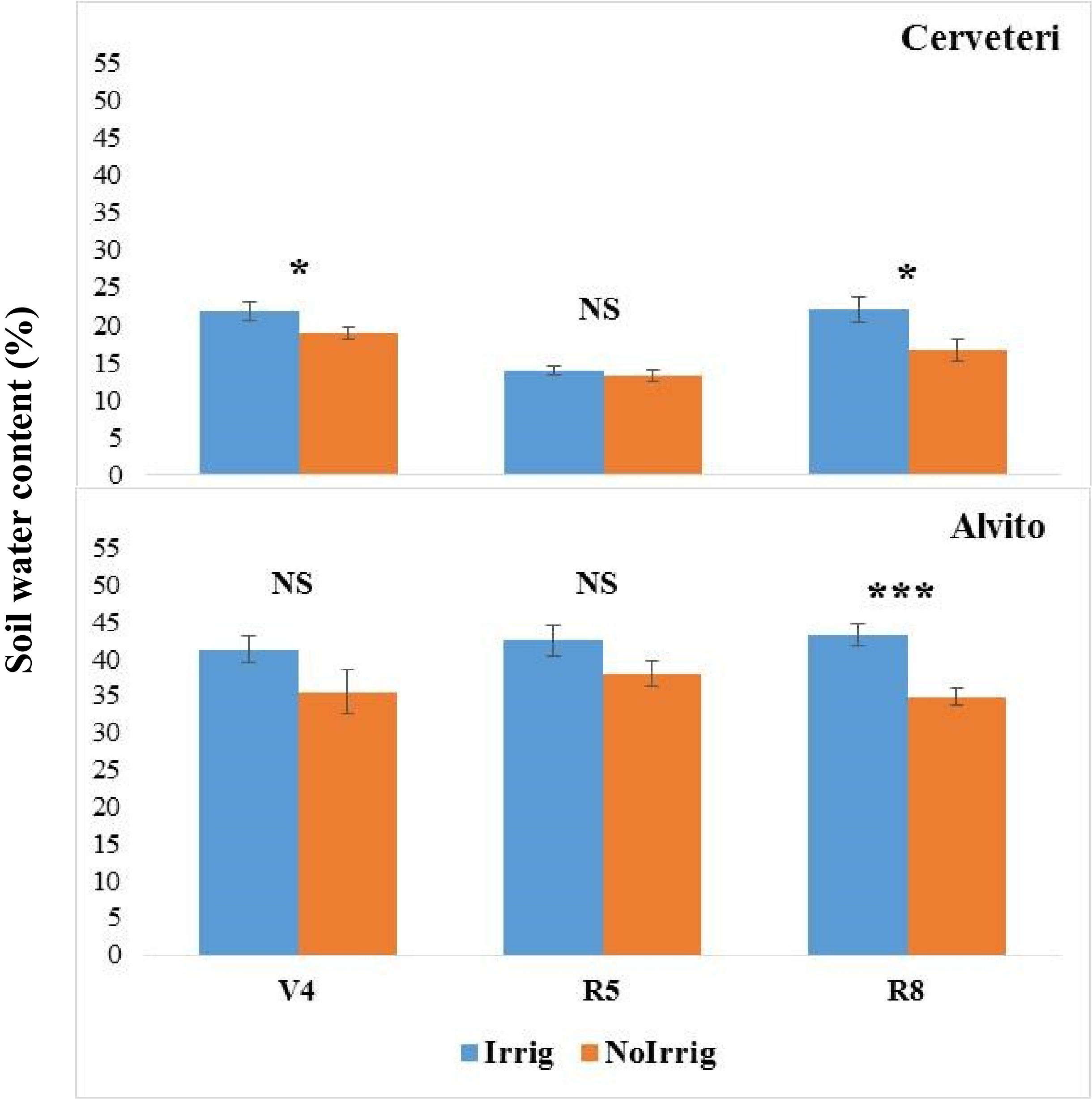

Figure S4. Volumetric soil water content (%) measured at the Cerveteri and the Alvito field sites, coincident with the three plants' phenological stages described in Section 2.2.5 of the main text. Asterisks denote statistically significant differences (*, $P<0.05$; ***, $P<0.001$; N=11) among irrigated (Irrig) and not irrigated (NoIrrig) plots within each phenological stage; NS, not statistically significant.

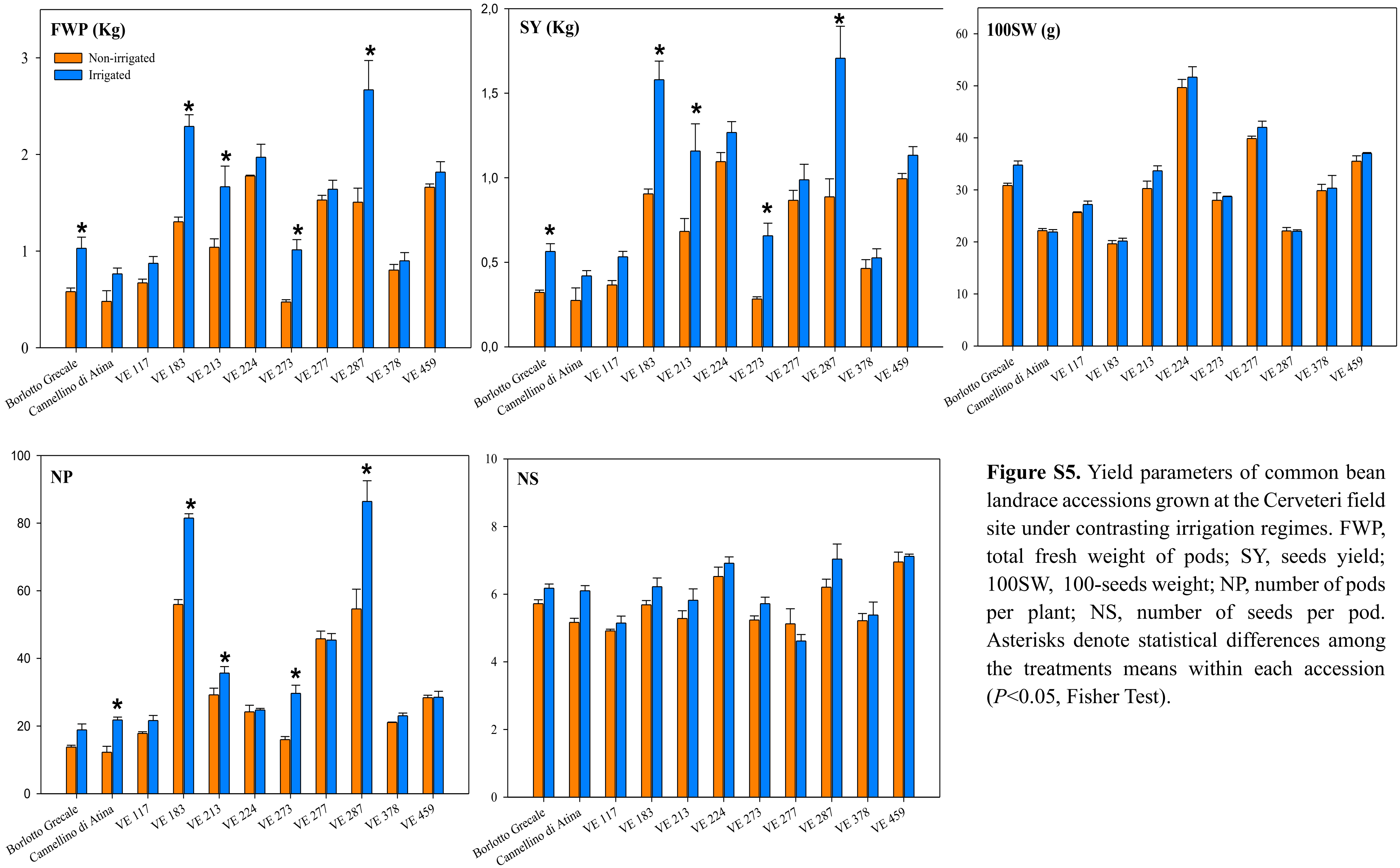

Figure S5. Yield parameters of common bean landrace accessions grown at the Cerveteri field site under contrasting irrigation regimes. FWP, total fresh weight of pods; SY, seeds yield; 100SW, 100-seeds weight; NP, number of pods per plant; NS, number of seeds per pod. Asterisks denote statistical differences among the treatments means within each accession ($P<0.05$, Fisher Test).

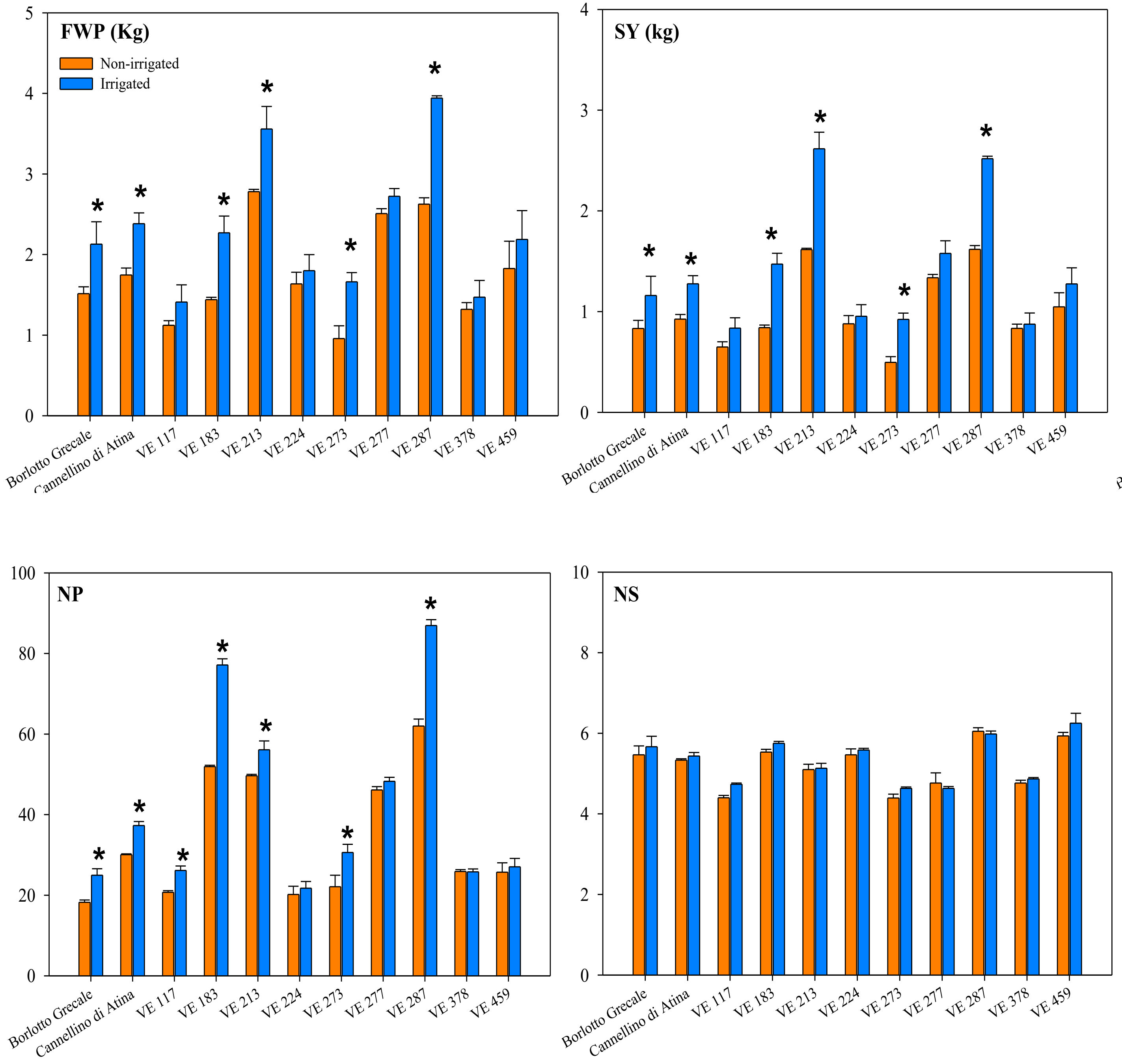

Figure S6. Yield parameters of common bean landrace accessions grown at the Alvito field site under contrasting irrigation regimes. Acronyms and statistics as in Fig. S5

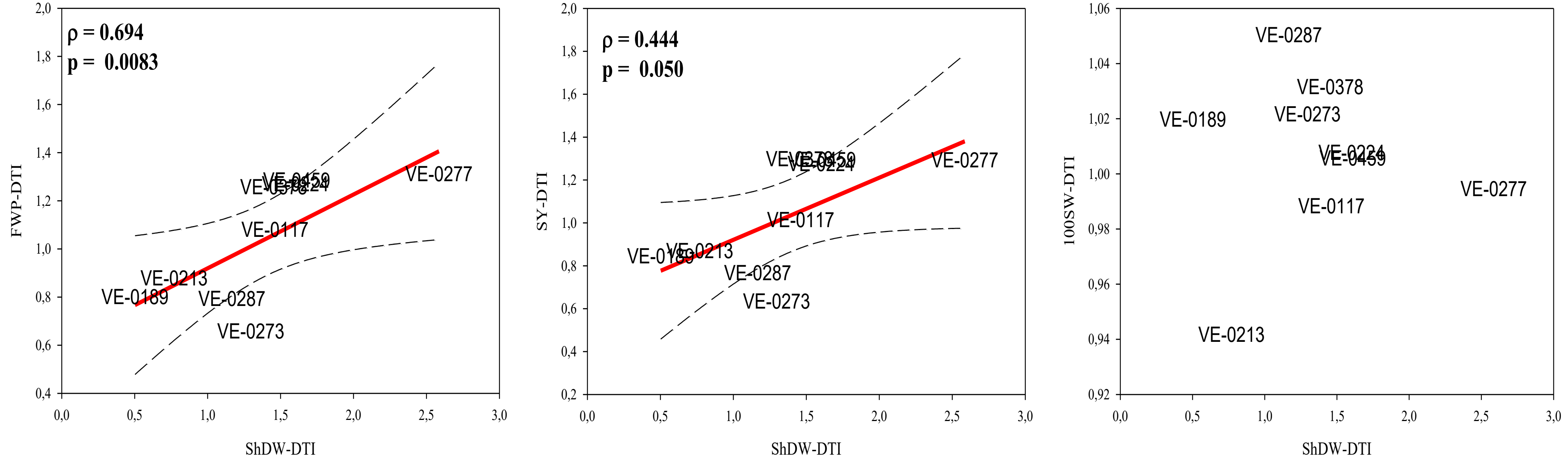

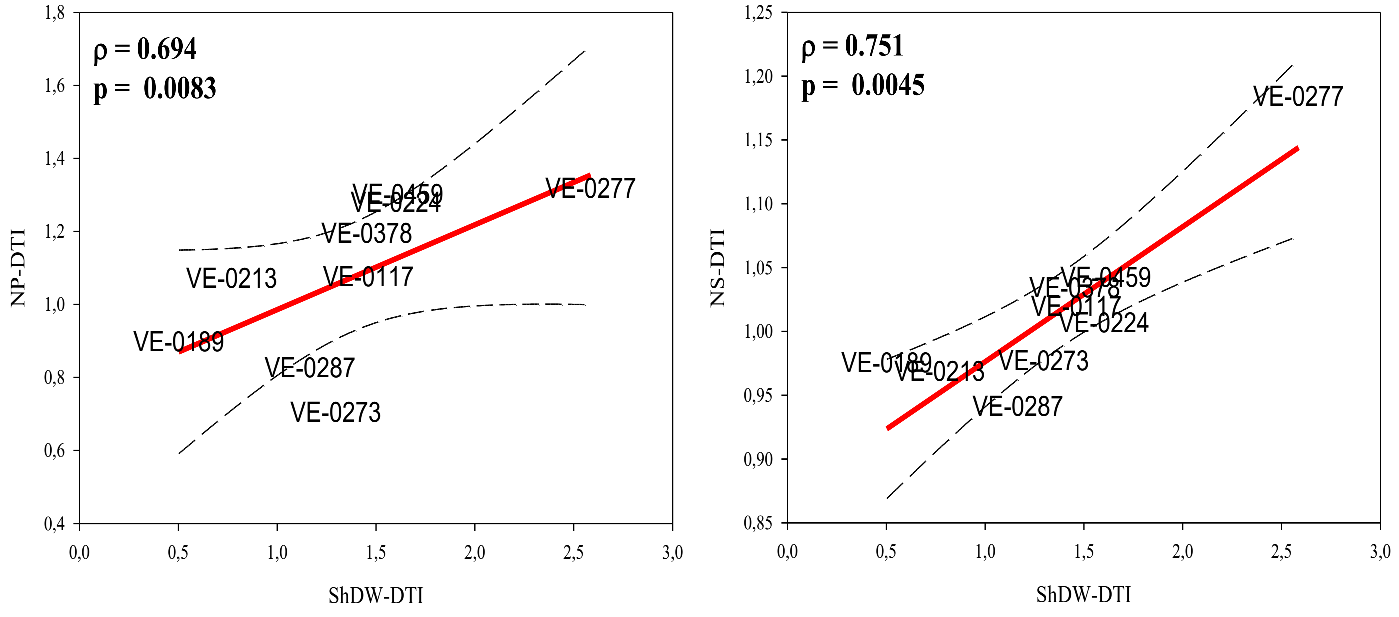

Figure S7. Correlation (Pearson test) between the drought tolerance index (DTI) based on the shoot dry weight (ShDW) of bean landrace accessions grown in growth chamber (pre-screening experiment; x axes) and the DTIs based on the yield parameters obtained from the same bean landrace accessions grown in the field at Cerveteri (y axes). Acronyms for yield parameters as in Fig. S5. ρ indicates the Spearmen coefficient of determination and p the probability value.

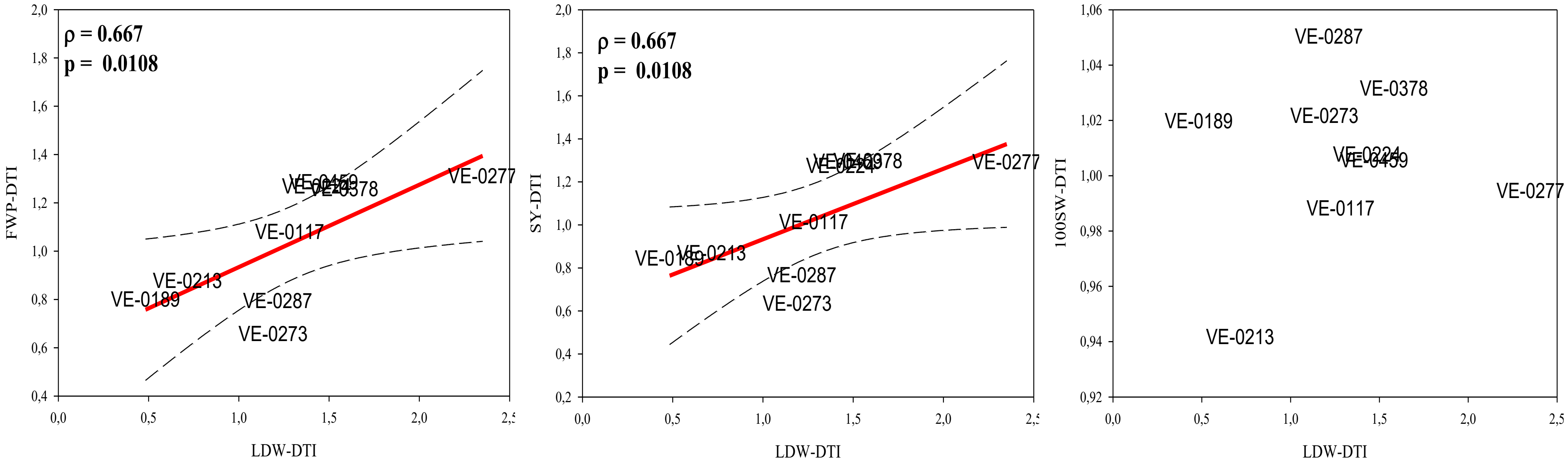

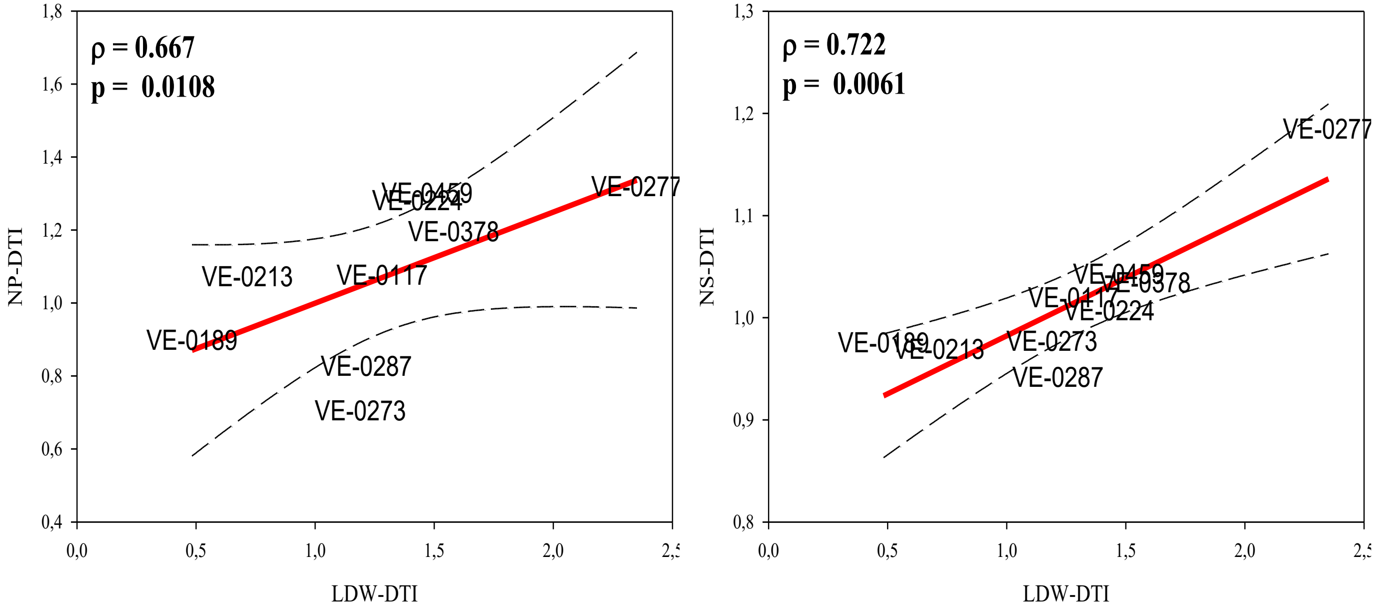

Figure S8. Correlation (Pearson test) between the drought tolerance index (DTI) based on the leaf dry weight (LDW) of bean landrace accessions grown in growth chamber (pre-screening experiment; x axes) and the DTIs based on the yield parameters obtained from the same bean landrace accessions grown in the field at Cerveteri (y axes). Acronyms for yield parameters as in Fig. S5. ρ indicates the Spearmen coefficient of determination and p the probability value.

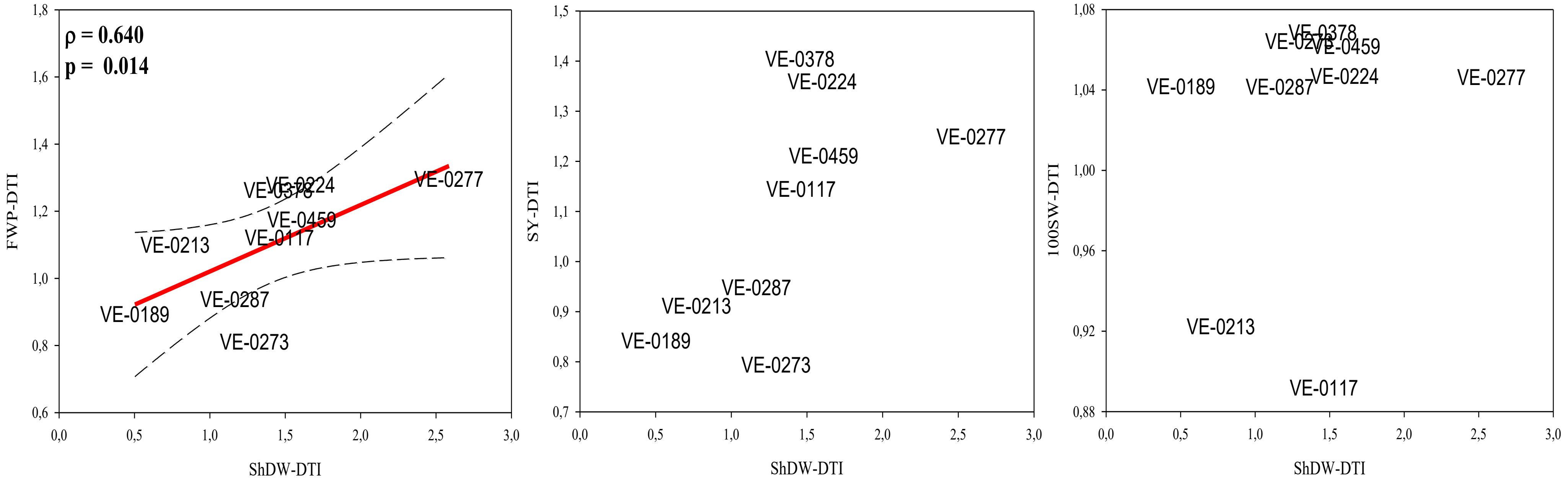

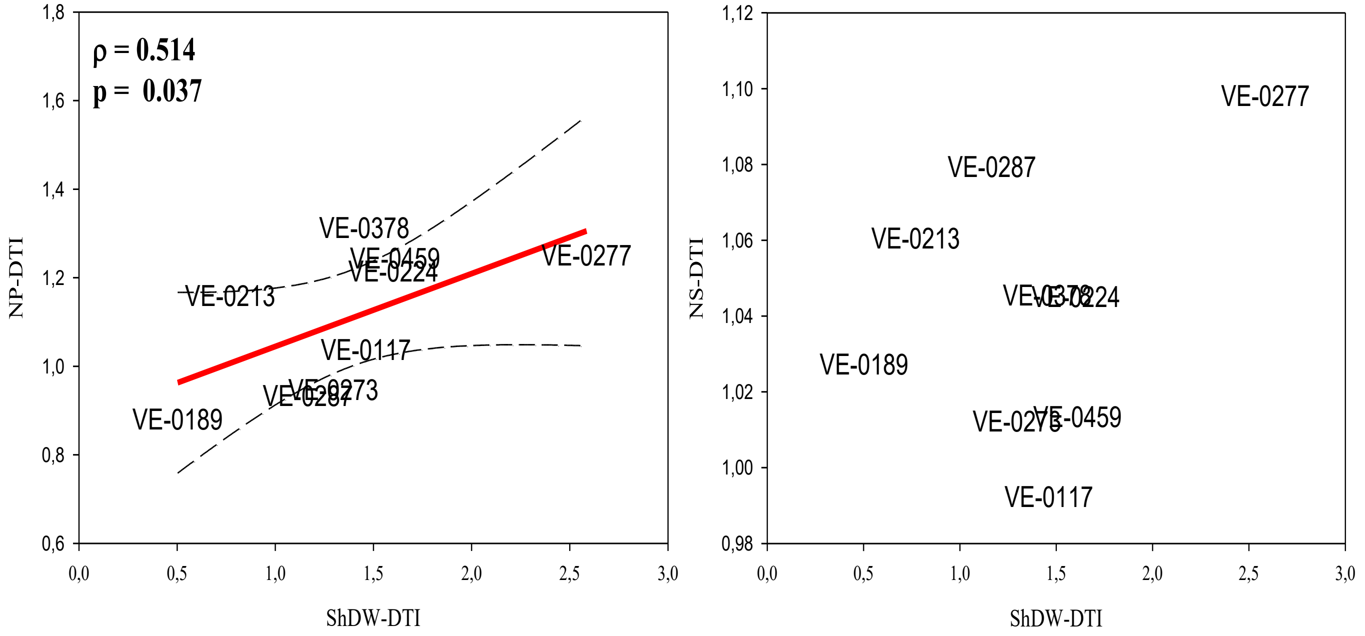

Figure S9. Correlation (Pearson test) between the drought tolerance index (DTI) based on the shoot dry weight (ShDW) of bean landrace accessions grown in growth chamber (pre-screening experiment; x axes) and the DTIs based on the yield parameters obtained from the same bean landrace accessions grown in the field at Alvito (y axes). Acronyms for yield parameters as in Fig. S5. ρ indicates the Spearmen coefficient of determination and p the probability value.

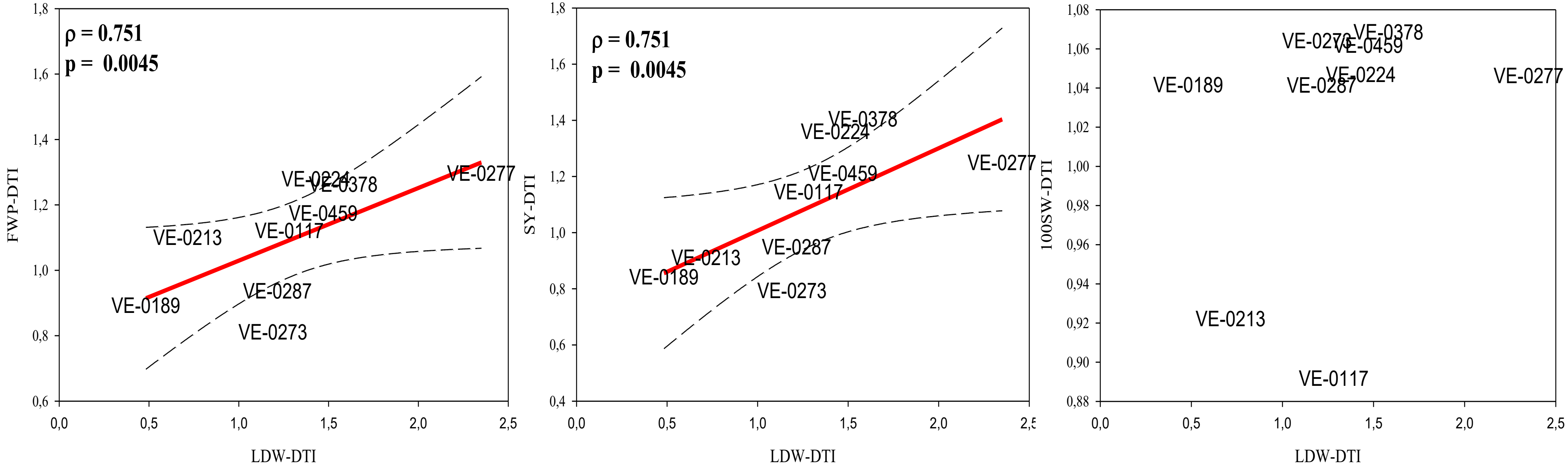

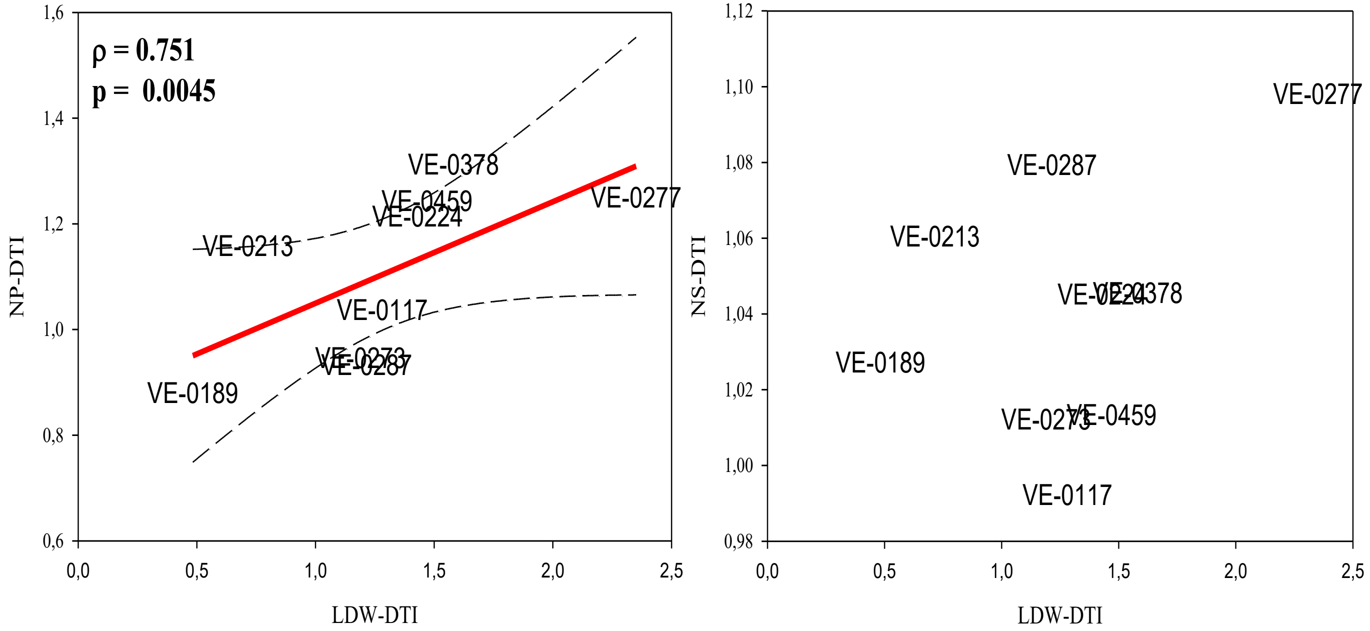

Figure S10. Correlation (Pearson test) between the drought tolerance index (DTI) based on the leaf dry weight (LDW) of bean landrace accessions grown in growth chamber (pre-screening experiment; x axes) and the DTIs based on the yield parameters obtained from the same bean landrace accessions grown in the field at Alvito (y axes). Acronyms for yield parameters as in Fig. S5. ρ indicates the Spearmen coefficient of determination and p the probability value.

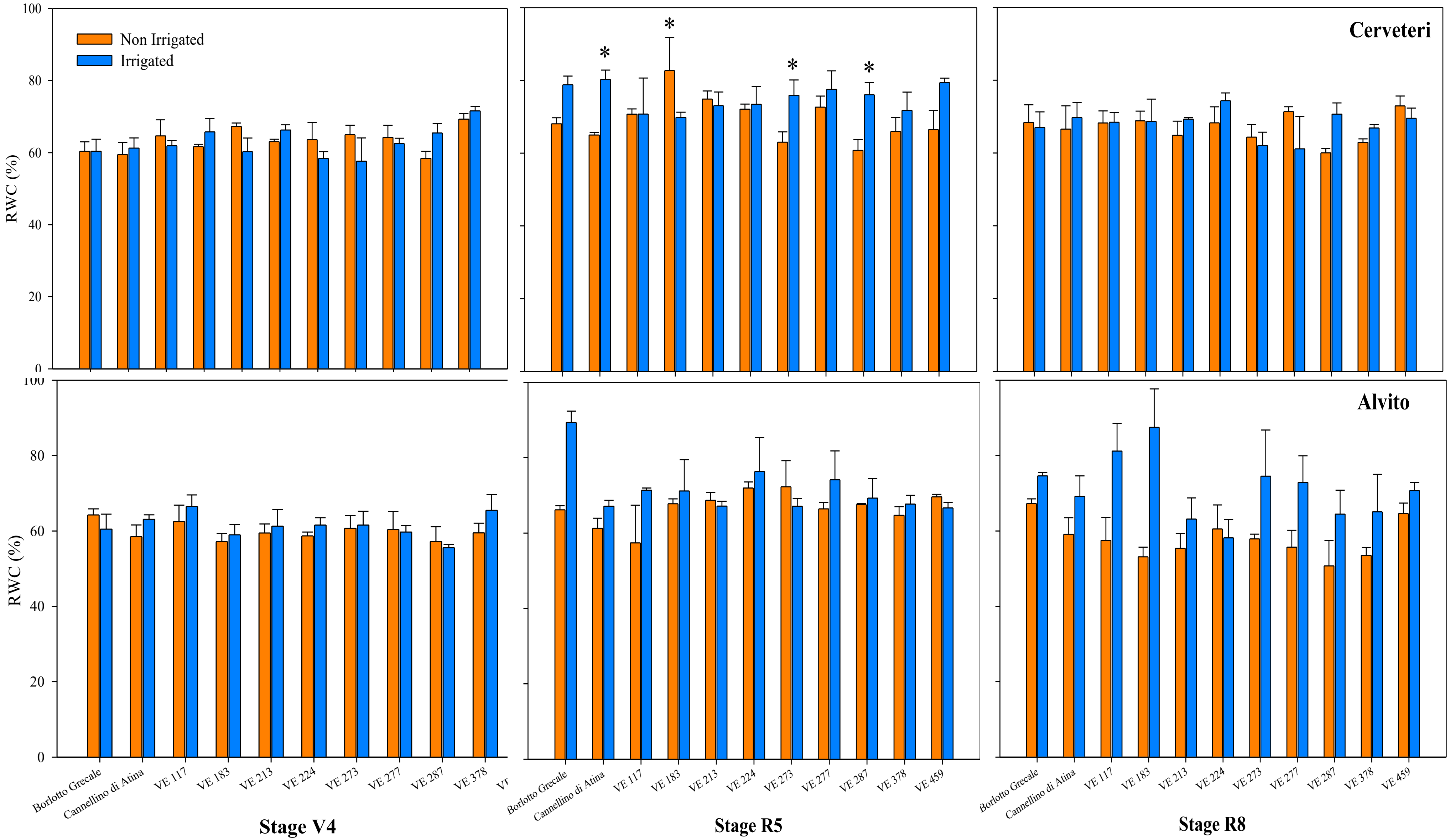

Figure S11. Leaf relative water content (RWC) in three phenological stages of bean landrace accessions grown under contrasting irrigation regimes at the two field sites of Cerveteri and Alvito. Asterisks denote statistically significant differences (*P*<0.05, Fisher Test) among the treatments.

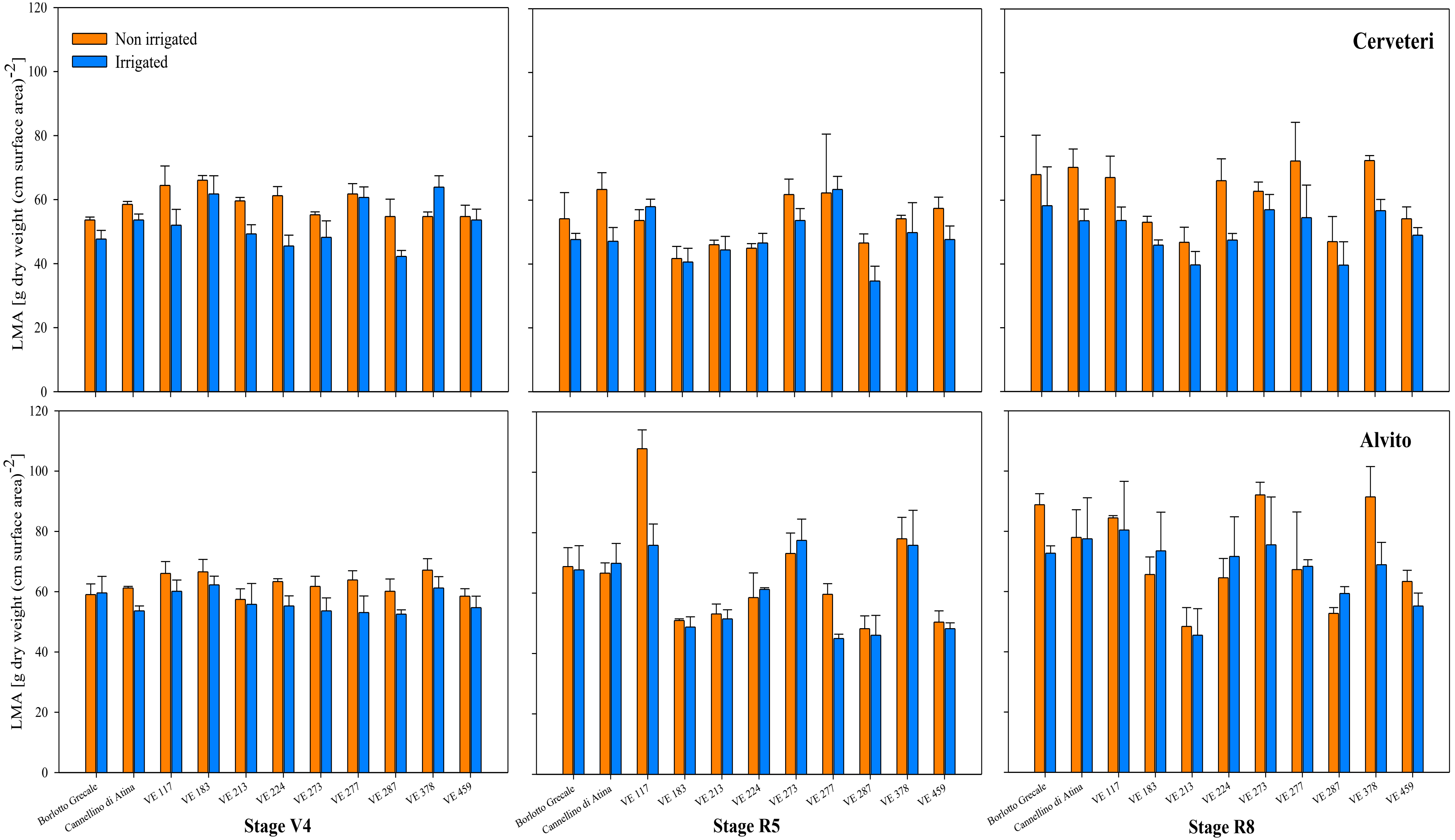

Figure S12. Leaf mass area (LMA) at three phenological stages of bean landrace accessions grown under contrasting irrigation regimes at the two field sites of Cerveteri and Alvito.

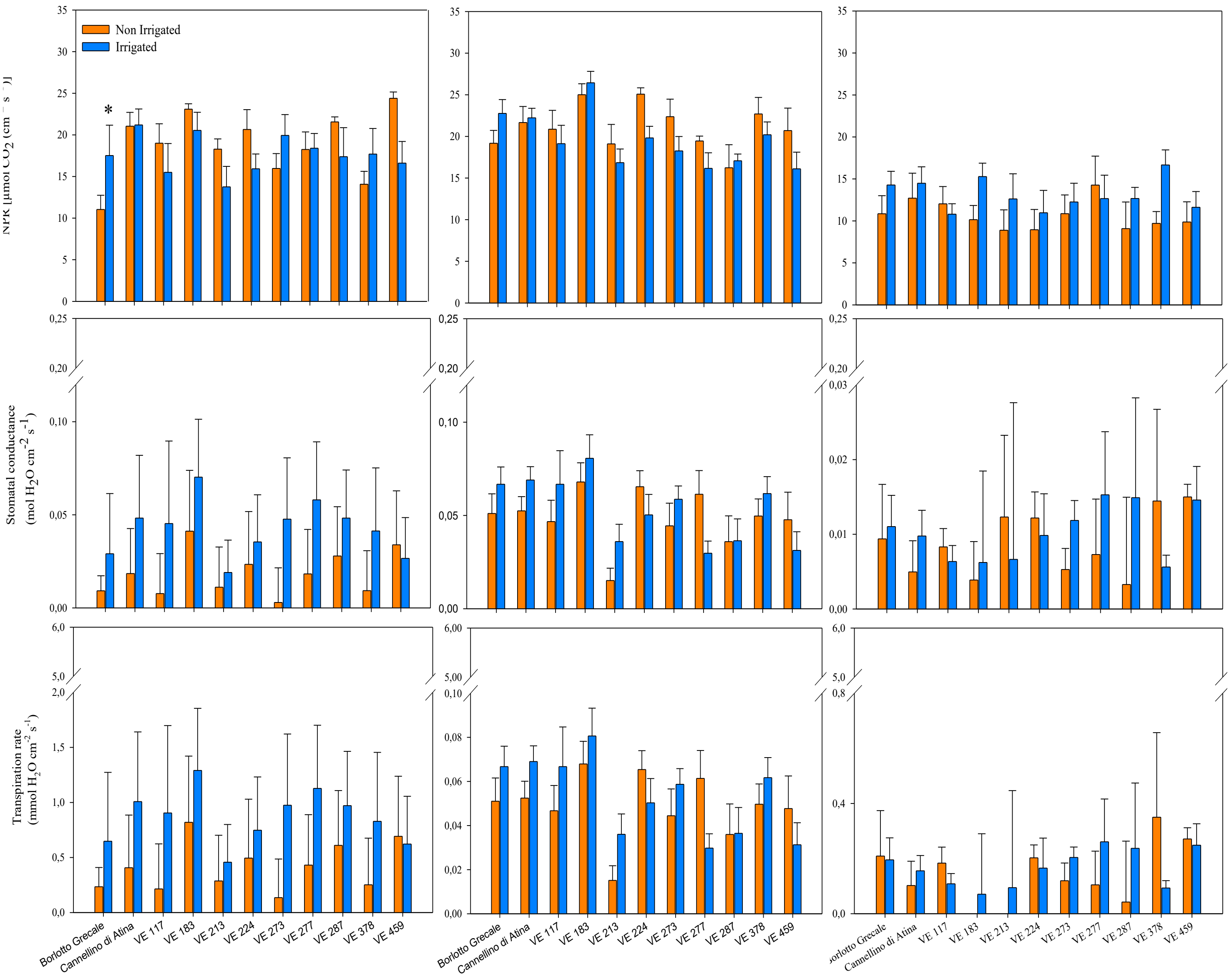

Figure S13. Net photosynthetic rate (NPR), stomatal conductance (SC) and transpiration rate (TR) in three phenological stages of bean landrace accessions grown under contrasting irrigation regimes at the Cerveteri field site. Asterisks denote statistically significant differences (*P*<0.05, Fisher Test) among the treatments.

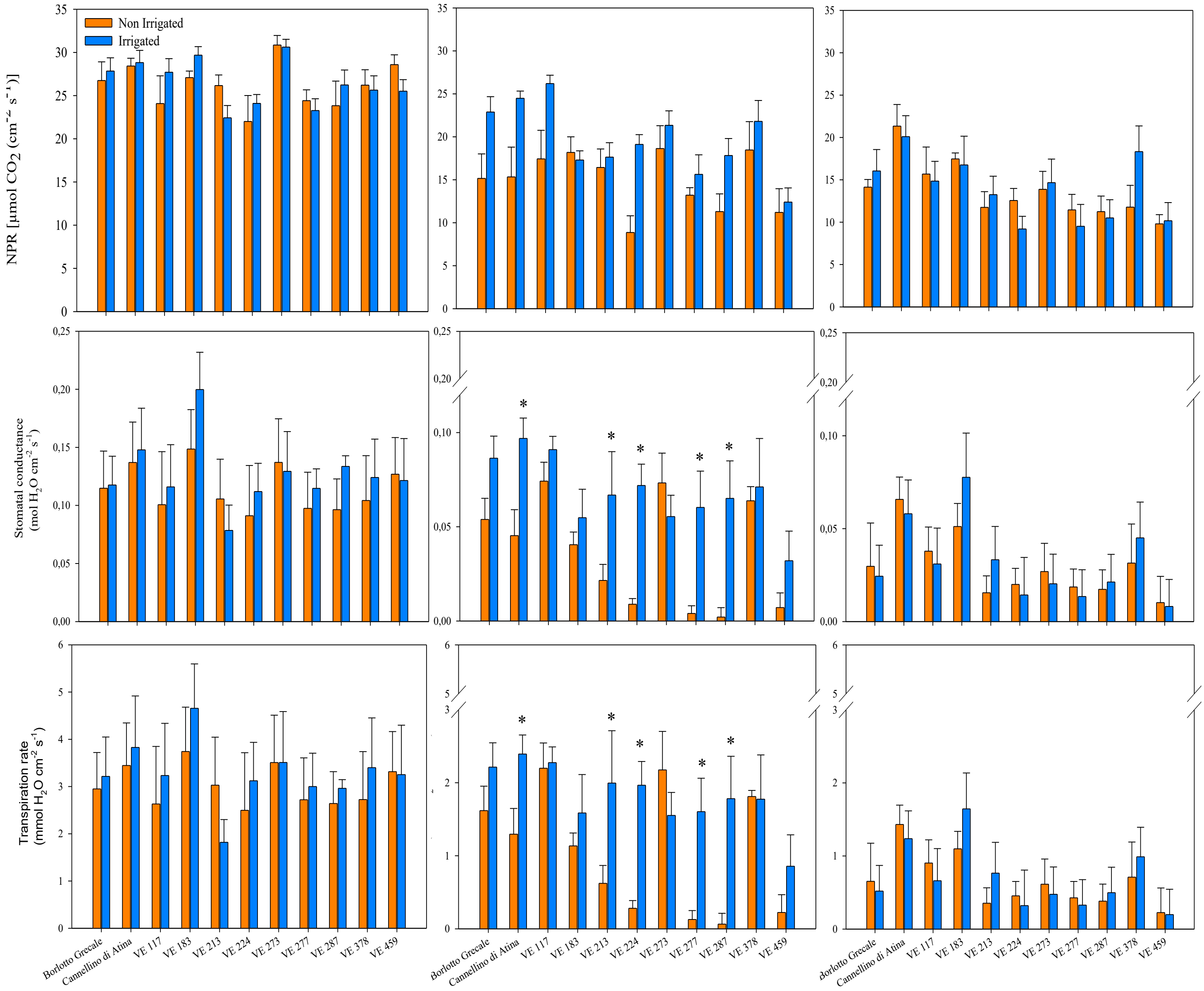

Figure S14. Net photosynthetic rate (NPR), stomatal conductance (SC) and transpiration rate (TR) in three phenological stages of bean landrace accessions grown under contrasting irrigation regimes at the Alvito field site. Asterisks denote statistically significant differences (*P*<0.05, Fisher Test) among the treatments

Supplementary Tables

Table S1. Common bean (*Phaseolus vulgaris* L.) landraces from the Lazio Region (Central Italy) screened for drought tolerance. ARSIAL, Lazio Regional Agency for Development and Innovation in Agriculture. Further details in Catarcione et al. [28].

ARSIAL code	Common name	Provenance	Province	Lat	Long	Elevation (m asl)	Landform	Growth habit	Customary water management	Gene pool
VE-0179	Ciavattone piccolo	Onano	Viterbo	424132N	114901E	510	hill	indeterminate	irrigated	Mesoamerican
VE-0191	Verdolino	Onano	Viterbo	424132N	114901E	510	hill	determinate	irrigated	Andean
VE-0183	Fagiolo del Purgatorio	Gradoli	Viterbo	423840N	115120E	470	hill	indeterminate	irrigated	Mesoamerican
VE-0277	Regina di Leonessa	Leonessa	Rieti	423407N	125736E	969	mountain	indeterminate	not irrigated	Andean
VE-0192	Verdolino di Montefiascone	Montefiascone	Viterbo	423225N	120213E	590	hill	determinate	irrigated	Andean
VE-0193	Occhietto di Montefiascone	Montefiascone	Viterbo	423225N	120213E	590	hill	determinate	irrigated	Andean
VE-0261	Borbontino	Borbona	Rieti	4231N	1308E	760	mountain	indeterminate	not irrigated	Andean
VE-0268	Pelone	Borbona	Rieti	4231N	1308E	760	mountain	indeterminate	not irrigated	Andean
VE-0273	Fagiolo di Corvaro	Corvaro	Rieti	4212N	1314E	732	mountain	determinate	irrigated	Andean
VE-0472	Giallo di Nazzareno	Borgorose	Rieti	4212N	1314E	732	mountain	determinate	not irrigated	Andean
VE-0473	Borlotto di Concetta	Borgorose	Rieti	4212N	1314E	732	mountain	determinate	not irrigated	Andean
VE-0243	Cioncone	Vallinfreda	Rome	4205N	1258E	874	mountain	indeterminate	irrigated	Andean
VE-0571	Fagiolina Arsolana	Arsoli	Rome	420226N	130104E	470	hill	indeterminate	irrigated	Mesoamerican
VE-0224	Regina di Marano Equo	Marano Equo	Rome	415938N	1301E	450	hill	indeterminate	irrigated	Andean
VE-0213	Cappellette di Vallepietra	Vallepietra	Rome	4156N	1314E	825	mountain	indeterminate	irrigated	Andean
VE-0215	Pallino di Vallepietra	Vallepietra	Rome	4156N	1314E	825	mountain	indeterminate	irrigated	Andean
VE-0222	Romanesco di Vallepietra	Vallepietra	Rome	4156N	1314E	825	mountain	indeterminate	irrigated	Andean
VE-0459	Fagiolo a Suricchio	Paliano	Frosinone	4148N	1303E	471	hill	indeterminate	not irrigated	Andean
VE-0378	Cannellino con la mosca	Casalvieri	Frosinone	4138N	1343E	380	plateau	determinate	not irrigated	Andean
VE-0110	Cannellino di Atina	Atina	Frosinone	4137N	1348E	481	hill	determinate	irrigated	Andean
VE-0287	Bottoncino di Terelle	Terelle	Frosinone	4133N	1347E	905	mountain	indeterminate	irrigated	Mesoamerican
VE-0117	Cannellino Rosso di Piumarola	Villa S. Lucia	Frosinone	4131N	1346E	393	plateau	determinate	irrigated	Andean
VE-0125	Cannellino Bianco di Piumarola	Villa S. Lucia	Frosinone	4131N	1346E	393	plateau	determinate	irrigated	Andean
VE-0128	Cannellino Grigio di Piumarola	Villa S. Lucia	Frosinone	4131N	1346E	393	plateau	determinate	irrigated	Andean

Table S2. Soil analysis before seeds sowing (0-30 cm depth) at the experimental field sites of Cerveteri and Alvito. CEC, cation exchange capacity; EC, electrical conductivity; OM, organic matter.

Parameter	Cerveteri	Judgement/rating	Alvito	Judgement/rating
**Texture class**		Sandy clay loam		Clay
**EC** (µS cm^{-1})	1,636	High	1,788	Very high
**pH** (H_2O)	6.50	Weakly acidic	6.97	Neutral
**CEC** [meq $(100g)^{-1}$]	20.00	Moderately high	38.90	High
**OM** (%)	1.61	Medium	3.25	Optimum
**Total N** (g kg^{-1})	0.97	Low	1.70	Well supplied
**C/N ratio**	9.64	Balanced - Normal mineralization	11.15	Balanced - Normal mineralization
**Available P** (µg P g^{-1})	12.76	Low	23.32	High
**K** [meq $(100g)^{-1}$]	0.89	Very high	0.48	Very high
**Ca** [meq $(100g)^{-1}$]	2.74	Low	1.39	Very low
**Na** [meq $(100g)^{-1}$]	72.23	Very high	7.58	Very high
**Mg** [meq $(100g)^{-1}$]	0.60	Low	0.22	Very low
**Mg/K** (meq/meq)	0.68	Low	0.46	Very low
**Fe** (mg Kg^{-1})	35.53	Medium	51.20	Optimum
**Mn** (mg Kg^{-1})	n.d.*		24.37	Low
**Zn** (mg Kg^{-1})	0,79	Very low	1.03	Low
**Cu** (mg Kg^{-1})	2,51	Optimum	3.10	High

* Not determined

Table S3. Pearson correlation analysis among different drought tolerance indexes calculated for common bean landrace accessions grown under controlled environmental conditions. Statistical significance at P<0.05 is denoted in bold. For acronyms, see Tables 1 and 2.

	SER-RC	LAER-RC	FoR-RC
ShDW- DTI	0.13032	**-0.5401**	**-0.56684**
ShDW-DTE	0.13032	**-0.5401**	**-0.56684**
LDW-DTI	0.117407	**-0.56994**	**-0.59738**
LDW-DTE	0.117407	**-0.56994**	**-0.59738**

Table S4. Comparison of rankings of different drought tolerance indexes calculated for common bean landrace accessions grown under controlled environmental conditions. Red or blue colors for landraces accessions' codes denote drought-tolerance or drought-susceptibility, respectively. For acronyms, see Tables 1 and 2.

SER-RC	LAER-RC	FoR-RC	ShDW- DTI	ShDW-DTE	LDW-DTI	LDW-DTE
VE-0193	VE-0222	VE-0459	VE-0277	VE-0277	VE-0277	VE-0277
VE-0192	VE-0378	VE-0378	VE-0459	VE-0459	VE-0378	VE-0378
VE-0571	VE-0472	VE-0277	VE-0224	VE-0224	VE-0459	VE-0459
VE-0179	VE-0273	VE-0110	VE-0117	VE-0117	VE-0224	VE-0224
VE-0378	VE-0128	VE-0125	VE-0378	VE-0378	VE-0117	VE-0117
VE-0222	VE-0117	VE-0215	VE-0273	VE-0273	VE-0287	VE-0287
VE-0459	VE-0459	VE-0213	VE-0110	VE-0110	VE-0273	VE-0273
VE-0213	VE-0110	VE-0222	VE-0287	VE-0287	VE-0110	VE-0110
VE-0472	VE-0277	VE-0261	VE-0571	VE-0571	VE-0571	VE-0571
VE-0273	VE-0224	VE-0243	VE-0473	VE-0473	VE-0243	VE-0243
VE-0183	VE-0125	VE-0273	VE-0268	VE-0268	VE-0473	VE-0473
VE-0261	VE-0287	VE-0117	VE-0243	VE-0243	VE-0472	VE-0472
VE-0287	VE-0213	VE-0192	VE-0128	VE-0128	VE-0268	VE-0268
VE-0243	VE-0571	VE-0472	VE-0192	VE-0192	VE-0192	VE-0192
VE-0224	VE-0215	VE-0128	VE-0472	VE-0472	VE-0128	VE-0128
VE-0268	VE-0192	VE-0268	VE-0215	VE-0215	VE-0222	VE-0222
VE-0128	VE-0261	VE-0224	VE-0222	VE-0222	VE-0215	VE-0215
VE-0117	VE-0473	VE-0287	VE-0125	VE-0125	VE-0125	VE-0125
VE-0125	VE-0268	VE-0193	VE-0213	VE-0213	VE-0213	VE-0213
VE-0473	VE-0243	VE-0571	VE-0261	VE-0261	VE-0191	VE-0191
VE-0110	VE-0193	VE-0473	VE-0191	VE-0191	VE-0261	VE-0261
VE-0277	VE-0191	VE-0191	VE-0193	VE-0193	VE-0193	VE-0193
VE-0215	VE-0179	VE-0183	VE-0183	VE-0183	VE-0183	VE-0183
VE-0191	VE-0183	VE-0179	VE-0179	VE-0179	VE-0179	VE-0179

Table S5. Comparative timetable of the key plant stages during the field experiments in Cerveteri and Alvito.

	Days after sowing	
	**Cerveteri**	**Alvito**
Sowing	0	0
Water shortage imposed	1	1
Stage V4 attained - 50% of the plants having their third trifoliate leaf unfolded*	31	36
Stage R5 attained - preflowering, beginning when the 50% of the plants show their first bud (determinate) or raceme (indeterminate)*	51	57
Stage R8 attained - pod filling, when the 50% of the plants begin to fill their first pod and the seeds inside the pod (10-12 cm in length) are discernible by sight and touch*	61	85
Pods agronomic maturity – Harvest	77	134

* CIAT, 1986

Table S6. Two-ways ANOVA on the yield parameters from common bean landrace accessions grown at the Cerveteri and the Alvito field sites under contrasting irrigation regimes (treatments). DF, degrees of freedom.

Site	Source	DF	F and P values				
			Total weight of pods (FWP)	***Total seed yield (SY)***	***100 seed weight (100-SW)***	***Number of pods per plant (NP)***	***Number of seeds per pod (NS)***
Cerveteri	*Accessions (A)*	10	54.43***	43.57***	148.55***	168.75***	16.61***
	Treatments (T)	1	96.36***	84.71***	10.42**	95.62***	13.53***
	Block	2	5.03*	NS	NS	4.33*	NS
	A x T	10	6.03***	5.03***	0.82^{NS}	12.56***	1.22^{NS}
Alvito	*Accessions (A)*	10	31.08***	49.74***	329.73***	320.32***	39.45***
	Treatments (T)	1	53.05***	92.91***	4.56*	164.27***	6.02*
	Block	2	NS	NS	NS	NS	NS
	A x T	10	20.33*	5.29***	3.36**	17.65***	0.69^{NS}

Statistics: *0.05 > P < 0.01; **0.01 > P < 0.001; ***0.001 > P; NS, not significant.

Table S7. Two-ways ANOVA on the relative water content of common bean landrace accessions grown at the Cerveteri and the Alvito field sites under contrasting irrigation regimes (treatments). DF, degrees of freedom.

Site	Source	DF	F and P values		
			Stage V4	***Stage R5***	***Stage R8***
Cerveteri	*Accessions (A)*	10	1.8497 NS	0.7317 NS	0.8240 NS
	Treatments (T)	1	0.1596 NS	10.5720**	0.3631 NS
	Block	2	NS	NS	NS
	A x T	10	1.2678 NS	2.1373*	1.0090 NS
Alvito	*Accessions (A)*	10	1.0158 NS	1.6137 NS	1.6177 NS
	Treatments (T)	1	2.9626 NS	6.6090*	32.5805***
	Block	2	NS	NS	5.4360**
	A x T	10	0.6858 NS	1.6319 NS	1.6047 NS

Statistics: *0.05 > P < 0.01; **0.01 > P < 0.001; ***0.001 > P; NS, not significant.

Table S8. Two-ways ANOVA on the leaf mass per area of common bean landrace accessions grown at the Cerveteri and the Alvito field sites under contrasting irrigation regimes (treatments). DF, degrees of freedom.

Site	Source	DF	F and P values		
			Stage V4	***Stage R5***	***Stage R8***
Cerveteri	*Accessions (A)*	10	3.7570**	2.9329**	2.9716**
	Treatments (T)	1	16.9329***	3.7249^{NS}	16.5374***
	Block	2	NS	NS	NS
	A x T	10	2.0510^{NS}	0.5990^{NS}	0.3165^{NS}
Alvito	*Accessions (A)*	10	1.3472^{NS}	12.7614***	3.0842***
	Treatments (T)	1	12.9508**	3.1634^{NS}	1.3614^{NS}
	Block	2	NS	NS	3.7836*
	A x T	10	0.3773^{NS}	1.6662^{NS}	0.6969^{NS}

Statistics: *0.05 > P < 0.01; **0.01 > P < 0.001; ***0.001 > P; NS, not significant.

Table S9. Two-ways ANOVA on the leaf gas exchange parameters (NPR, net photosynthetic rate; SC, stomatal conductance; TR, transpiration rate) of common bean landrace accessions grown at the Cerveteri field site under contrasting irrigation regimes (treatments). DF, degrees of freedom.

Parameter	Source	DF	F and P values		
			Stage V4	***Stage R5***	***Stage R8***
NPR	*Accessions (A)*	10	2.6122**	4.3373***	1.5898 NS
	Treatments (T)	1	1.7956 NS	4.4088*	20.4517***
	Block	2	11.3831***	3.9058*	36.1475***
	A x T	10	2.3219*	1.2861 NS	1.1251 NS
SC	*Accessions (A)*	10	0.3143 NS	3.1393**	0.6680 NS
	Treatments (T)	1	4.2179*	0.9574 NS	3.1001 NS
	Block	2	NS	NS	5.6736**
	A x T	10	0.1576 NS	1.3491 NS	0.5535 NS
TR	*Accessions (A)*	10	0.2614 NS	3.2543***	0.6980 NS
	Treatments (T)	1	4.1956*	0.009 NS	1.0094 NS
	Block	2	NS	NS	3.3808*
	A x T	10	0.1301 NS	1.0766 NS	0.6143 NS

Statistics: *0.05 > P < 0.01; **0.01 > P < 0.001; ***0.001 > P; NS, not significant.

Table S10. Two-ways ANOVA on the leaf gas exchange parameters (NPR, net photosynthetic rate; SC, stomatal conductance; TR, transpiration rate) of common bean landrace accessions grown at the Alvito field site under contrasting irrigation regimes (treatments). DF, degrees of freedom.

**Parameter**	**Source**	**DF**	**F and P values**		
			Stage V4	***Stage R5***	***Stage R8***
NPR	*Accessions (A)*	10	4.5072***	5.2988***	4.2890***
	Treatments (T)	1	0.1963 NS	32.2057***	0.0467 NS
	Block	2	12.6597***	12.9531***	NS
	A x T	10	0.3047 NS	1.8876 NS	0.6641 NS
SC	*Accessions (A)*	10	4.2007***	4.8877***	2.1576*
	Treatments (T)	1	3.9886*	36.4068***	0.0499 NS
	Block	2	189.3876***	NS	NS
	A x T	10	1.0216 NS	2.3410*	0.2643 NS
TR	*Accessions (A)*		3.3095**	4.4238***	2.1962*
	Treatments (T)		3.2618 NS	26.4537***	0.0508 NS
	Block		284.1817***	NS	NS
	A x T		1.2052 NS	2.6141**	0.2691 NS

Statistics: *0.05 > P < 0.01; **0.01 > P < 0.001; ***0.001 > P; NS, not significant.

Table S11. Coefficients of variation of different traits [related to yield (Y), physiology (P), or morphology (M)] measured at three different growth stages, namely V4, R5, and R8, in common bean landrace accessions grown at two different field sites (C, Cerveteri; A, Alvito) under contrasting water regimes, namely irrigated (Irrig) or non-irrigated (NoIrrig). Coefficients of variation <10% (low genetic variability), 10-20% (medium genetic variability) or >20% (high genetic variability) are denoted in normal, italics or bold, respectively. Acronyms used for traits as in Table 5.

Trait	Type	Stage	Site	Irrig	NoIrrig
100SW	Y	R8	C	**29**	**46**
FWP	Y	R8	C	**43**	**48**
NP	Y	R8	C	**62**	**28**
NS	Y	R8	C	*14*	**54**
SY	Y	R8	C	**48**	*13*
NPR	P	R5	C	**24**	**28**
NPR	P	V4	C	**36**	**24**
NPR	P	R8	C	**38**	**47**
SC	P	R5	C	**58**	**52**
SC	P	V4	C	**64**	**55**
SC	P	R8	C	**71**	**85**
TR	P	R5	C	**55**	**50**
TR	P	R8	C	**70**	**51**
TR	P	V4	C	**55**	**68**
RWC	P	V4	C	9	8
RWC	P	R5	C	10	*12*
RWC	P	R8	C	10	9
LMA	M	V4	C	*16*	10
LMA	M	R5	C	*20*	23
LMA	M	R8	C	*20*	22
FWP	Y	R8	A	**35**	**36**
SY	Y	R8	A	**36**	**44**
100SW	Y	R8	A	**28**	**27**
NP	Y	R8	A	**45**	**52**
NS	Y	R8	A	*11*	*11*
NPR	P	V4	A	*15*	*19*
NPR	P	R5	A	**27**	**44**
NPR	P	R8	A	**48**	**40**
SC	P	V4	A	**56**	**73**
SC	P	R5	A	**60**	**70**
SC	P	R8	A	**69**	**89**
TR	P	R5	A	**61**	**74**
TR	P	V4	A	**64**	**72**
TR	P	R8	A	**64**	**86**
RWC	P	V4	A	8	8
RWC	P	R5	A	*13*	*11*
RWC	P	R8	A	*19*	*13*
LMA	M	V4	A	*12*	9.2
LMA	M	R5	A	**25**	**28**
LMA	M	R8	A	**27**	**26**
